# Regularized dynamical decoupling noise spectroscopy – a decoherence descriptor for radicals in glassy matrices†

**DOI:** 10.1039/d1cp03103a

**Published:** 2021-09-23

**Authors:** Janne Soetbeer, Luis Fábregas Ibáñez, Zachariah Berkson, Yevhen Polyhach, Gunnar Jeschke

**Affiliations:** Laboratory of Physical Chemistry, ETH Zürich Vladimir-Prelog-Weg 2 CH-8049 Zürich Switzerland janne.soetbeer@phys.chem.ethz.ch

## Abstract

Decoherence arises from a fluctuating spin environment, captured by its noise spectrum *S*(*ω*). Dynamical decoupling (DD) with *n* π pulses extends the dephasing time if the associated filter function attenuates *S*(*ω*). Inversely, DD noise spectroscopy (DDNS) reconstructs *S*(*ω*) from DD data by approximating the filters pass band by a *δ*-function. This restricts application to qubit-like spin systems with inherently long dephasing times and/or many applicable pulses. We introduce regularized DDNS to lift this limitation and thereby infer *S*(*ω*) from DD traces of paramagnetic centers in glassy *o*-terphenyl and water–glycerol matrices recorded with *n* ≤ 5. For nitroxide radicals at low temperatures, we utilize deuteration to identify distinct matrix- and spin center-induced spectral features. The former extends up to a matrix-specific cut-off frequency and characterizes nuclear spin diffusion. We demonstrate that rotational tunneling of intramolecular methyl groups drives the latter process, whereas at elevated temperatures *S*(*ω*) reflects the classical methyl group reorientation. Ultimately, *S*(*ω*) visualizes and quantifies variations in the electron spins couplings and thus reports on the underlying spin dynamics as a powerful decoherence descriptor.

## Abstract 2

A noise spectrum captures the decoherence-inducing spin environment fluctuations and thus provides a quantitative decoherence description.

## Graphical abstract

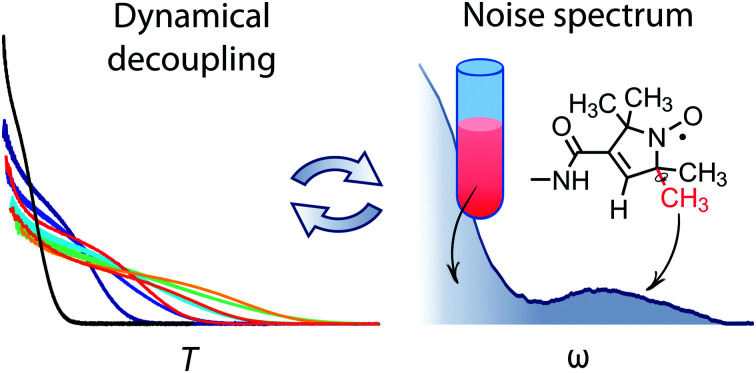

## 1 Introduction

**Fig. 1:**
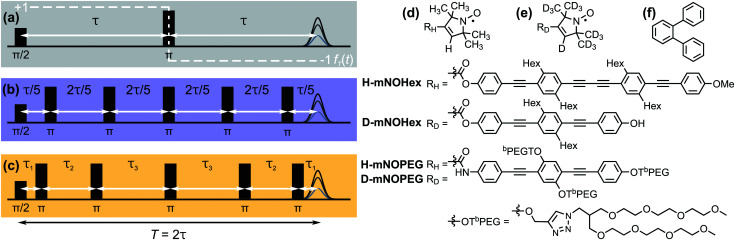
Representative dynamical decoupling pulse sequences for a total sequence length *T* = 2*τ*. (a) Hahn with the associated time-domain filter function *f*_*T*_(*t*) (white, dashed), (b) CP *n* = 5 and (c) Uhrig *n* = 5. Chemical structures of (d) protonated and (e) deuterated *gem*-dimethyl-nitroxides and the glass former (f) *o*-terphenyl.

Phase memory loss of electron spins constitutes a major obstacle to both quantum information and pulsed electron paramagnetic resonance (EPR) applications. Such loss, which is not necessarily caused by irreversible relaxation, can be considered as decoherence. Following spin excitation, the time until coherence is lost controls the available time window for spin manipulation. This decoherence time limits the feasible complexity of the applied operations, and determines the achievable spectral resolution and sensitivity. Dynamical decoupling (DD) is a control technique^1^ designed to overcome these limitations by applying multiple π pulses that refocus the decoherence-inducing system-environment interactions. The Carr-Purcell (CP) scheme^2^ realizes this principle by a uniform distribution of *n* π pulses over the total sequence length *T* (Fig. 1(b) for *n* = 5). It thereby extends the Hahn echo^3^ as the simplest refocusing experiment with *n* = 1 (Fig. 1(a)), while the Uhrig^4^ sequence relies on optimized delays (Fig. 1(c) for *n* = 5). Thus, each of these control schemes exerts a specific time-domain modulation on the central (electron) spin that translates into a frequency-domain filter function by Fourier transform. This formalism^4,5^ views dephasing under DD as a filter acting on the spectral density of the system-environment interactions, the noise spectrum. DD noise spectroscopy (DDNS) inverts this idea by inferring the underlying noise spectrum from decoherence measurements under DD control.^6–9^ This spectral reconstruction technique has characterized the spin environment of various qubit implementations including trapped ions,^10^ superconducting circuits,^6^ semiconductor quantum dots,^11^ phosphor donor electrons in silicon^12^ and nitrogen-vacancy (NV) centers in diamond.^13^ Most of these and similar DDNS applications approximate the frequency-domain filter expression by a series of *δ*-functions.^7,14^ The simplest reconstruction method is based on the leading term of the filter expression centered at a fundamental frequency to scan the noise spectrum by varying the interpulse settings and/or sequence length.^9^ An alternative scheme considers contributions at higher harmonics and determines the noise spectrum by building an invertible system of linear equations from a set of DD measurements.^8^ Both approaches are only applicable in the limit of many pulses, for long sequence lengths *T*^7^ or many DD cycles.^14^ Typical EPR applications to chemical and biological systems of current interest do not satisfy these conditions due to decoherence times in the microsecond range and limitations in the number of applicable pulses. While narrow-lined qubits allow for tens^8,11^ to hundreds^6,10,13^ to thousands^12^ control operations, the large spectral width of organic radicals or metal ions compared to available excitation bandwidths requires extensive phase cycling to isolate the desired coherence pathway and also leads to a progressive echo intensity loss with increasing *n*.^15,16^ We therefore introduce a new DDNS method that relies on the analytical filter functions and leverages regularization to stabilize the otherwise ill-conditioned inverse problem of inferring the noise spectrum from DD data. This way, refocusing schemes featuring only a few pulses can access the underlying noise spectrum by our regularized approach that enforces non-negativity and smoothness.

We utilize our CP and Uhrig DD measurements of nitroxide radicals (Fig. 1(d) and (e)) in *o*-terphenyl^15^ (OTP, Fig. 1(f)) and water–glycerol glass^16^ to demonstrate our procedure for 1 to 5 π pulses. In both solvents, the low-temperature (40 K) noise spectra reveal two separate features, which we assign by deuteration to spin center- and matrix induced processes. The noise spectra reflect temperature, solvent, concentration and isotope changes; thereby we monitor and visualize methyl group dynamics from the quantum to the classical regime, nuclear and electron spin driven diffusion as well as deuteration effects. Furthermore, we identify properties of the noise spectrum that rationalize the previously reported DD characteristics.^15,16^ These include the observation of a fast and a slow decoherence pathway, a linear increase of the dephasing time with *n*, and superior Uhrig decoupling in protonated solvents compared to CP refocusing.

The here investigated glassy matrix represents a common spin environment for application work, including dynamic nuclear polarization (DNP) experiments, while its complex bath dynamics are currently not well understood. Conventionally, the decoherence process of an electron spin in such matrices is probed by the Hahn experiment and assessed in terms of a dephasing time *T*_m_ and a stretch parameter *ξ* that together model the observed echo decay. Compared to this conventional decoherence analysis, we show that the noise spectrum provides a far more detailed characterization of the spin environment and thereby functions as a tangible decoherence descriptor.

## 2 Results and discussion

### 2.1 Noise spectroscopy and regularization

The standard DDNS formalism is based on three assumptions, namely pure dephasing, perfect refocusing pulses and Gaussian noise.^9^ Neglecting longitudinal relaxation, decoherence after initiation by the first π/2 pulse occurs due to spin environment interactions captured by stochastic modulation *b*_*z*_(*t*)*Ŝ*_*z*_ of the field parallel to the quantization axis. The pure dephasing mechanism thus describes variation in the precession frequency of the central spin, induced exclusively by the variable *b*_*z*_(*t*). Instead, the transverse elements *b*_*x*_(*t*) and *b*_*y*_(*t*) of this stochastic variable have to be considered in case longitudinal relaxation occurs during the spin evolution time. A DD sequence with *n* ideal and instantaneous π pulses act as a time-domain filter function *f*_*T*_(*t*) defined by the pulse positions *t*_*k*_ (with *t*_0_ = 0 and *t*_*n*+1_ = *T*) 
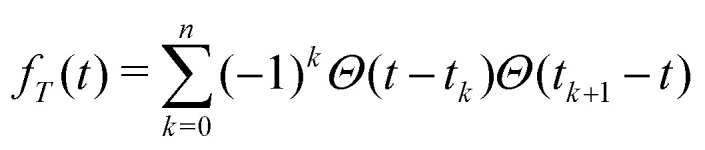
. Where *Θ*(*t*) stands for the Heaviside function, so that *f*_*T*_(*t*) switches between +1 and −1 at *t* = *t*_*k*_ as illustrated for the Hahn sequence in Fig. 1(a). In case of pure dephasing, the overall phase accumulation under DD control 
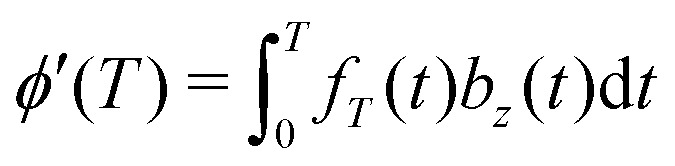
 leads to the decoherence function 
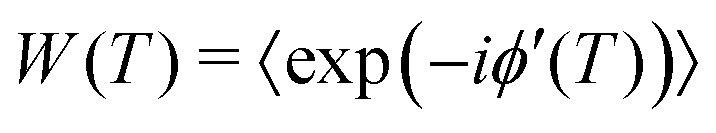
, obtained after averaging (〈…〉) over all *b*_*z*_(*t*) realizations. Assuming that *b*_*z*_(*t*) fulfills Gaussian properties, the average can be conveniently rewritten^9^ as 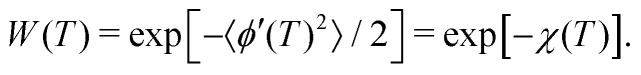
‡‡Note, that the Gaussian noise assumption simplifies the expression for the decoherence function *W*(*T*) significantly as the average can now be performed over the argument of the exponential function. For a classical, stationary noise process, we express the introduced attenuation function *χ*(*T*) in terms of the auto-correlation *C*(*t*_1_ − *t*_2_) = 〈*b*_*z*_(*t*_1_)*b*_*z*_(*t*_2_)〉, resulting in1
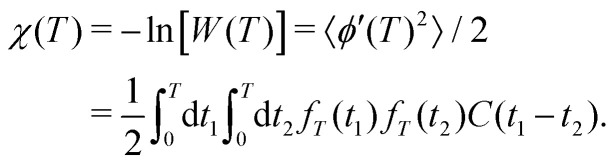


**Fig. 2:**
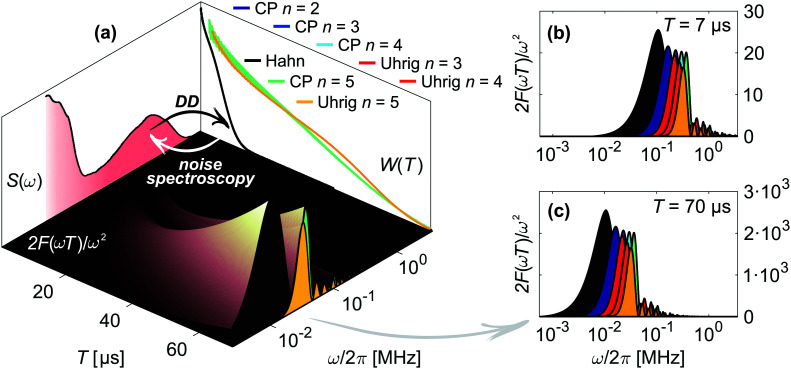
Concept of dynamical decoupling noise spectroscopy. (a) The filter function 2*F*(*ωT*)/*ω*^2^ of a DD pulse sequence (inner surface) connects the associated decoherence measurement *W*(*T*) with the noise spectrum *S*(*ω*), illustrated here for Hahn (black), CP (cyan) and Uhrig (yellow) *n* = 5 of 20 μM H-mNOHex in OTP. The displayed *W*(*T*) and *S*(*ω*) do not correspond to projections. Instead this short-hand representation depicts that a DD measurement (black arrow) probes *S*(*ω*) in the frequency range determined by the applied filter function. Inversely, noise spectroscopy infers the underlying *S*(*ω*) from *W*(*T*) (white arrow) as each time point *T* of *W*(*T*) arises from *S*(*ω*) that passes through the experiment-specific filter function for a sequence length *T*. The latter is shown in (b)–(c) for illustrative sequence lengths of (b) *T* = 7 μs and (c) *T* = 70 μs and for all here employed DD experiments (color-coded as in (a)). The Hahn, CP and Uhrig *n* = 5 filter functions in (b)–(c) thus correspond to vertical cuts through the three-dimensional filter representation in (a). From both representations it is evident that the main pass band of the DD filter functions shifts to smaller frequencies for longer *T*. Hence, applying a DD sequence with increasing *T* scans *S*(*ω*) from its high to its low-frequency region.

Fourier transformation establishes the DDNS equation2
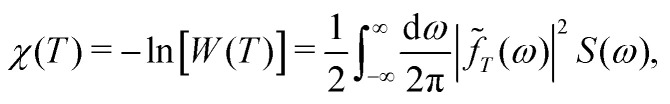
which relates *W*(*T*) to the noise spectrum *S*(*ω*) passed through a frequency-domain filter function |*f̃*_*T*_(*ω*)|^2^ = 2*F*(*ωT*)/*ω*^2^ as illustrated in Fig. 2.

Experimentally, a decoherence measurement determines *n*_*T*_ integrated echo intensities at times *T*_*i*_, forming a vector ***W*** with entries *W*_*i*_ = *W*(*T*_*i*_). In DDNS terms, the applied refocusing sequence of total length *T*_*i*_ is associated with an *n*_*ω*_-element filter vector at discrete frequencies *ω*_*j*_, all separated by an increment Δ*ω*. The entire acquisition at *n*_*T*_ time points *T*_*i*_ therefore forms an *n*_*T*_ × *n*_*ω*_ filter kernel matrix **F** with elements *F*_*i*,*j*_ = *F*(*ω*_*j*_*T*_*i*_)Δ*ω*/π*ω*_*j*_^2^, that probes the *n*_*ω*_ elements *S*_*j*_ = *S*(*ω*_*j*_) of the noise vector ***S*** (see Fig. 2(a)). The discretized form of eqn (2) thus reads ***χ*** = −ln(***W***) = **F*****S***. The large condition number of **F** prohibits an accurate and stable noise spectrum reconstruction *via* the direct inversion ***S*** = **F**^−1^***χ***. Instead, we reformulate this problem and infer ***S*** from DD data *via* a regularized least squares minimization3
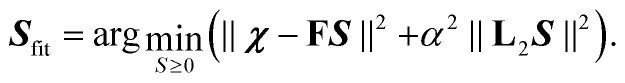


The first term encourages agreement between the decoherence model ***χ***_fit_ = **F*****S***_fit_ and the decoherence measurement ***χ*** = −ln(***W***), whereas the second term imposes smoothness onto the solution ***S***_fit_ by employing the second-order discrete differential operator matrix **L**_2_. The large number of coupled spins contributing to the decoherence-inducing spin environment justifies this enforcement of smoothness, and the non-negativity constraint (***S*** ≥ 0) arises as a mathematical property of a spectral density function. Both constraints allow the inference of a unique solution for a given regularization parameter *α*. We rely on the Akaike information criterion (AIC)^17^ to select an *α* value that balances the goodness of fit with the complexity of the model, providing a parsimonious solution.

**Fig. 3:**
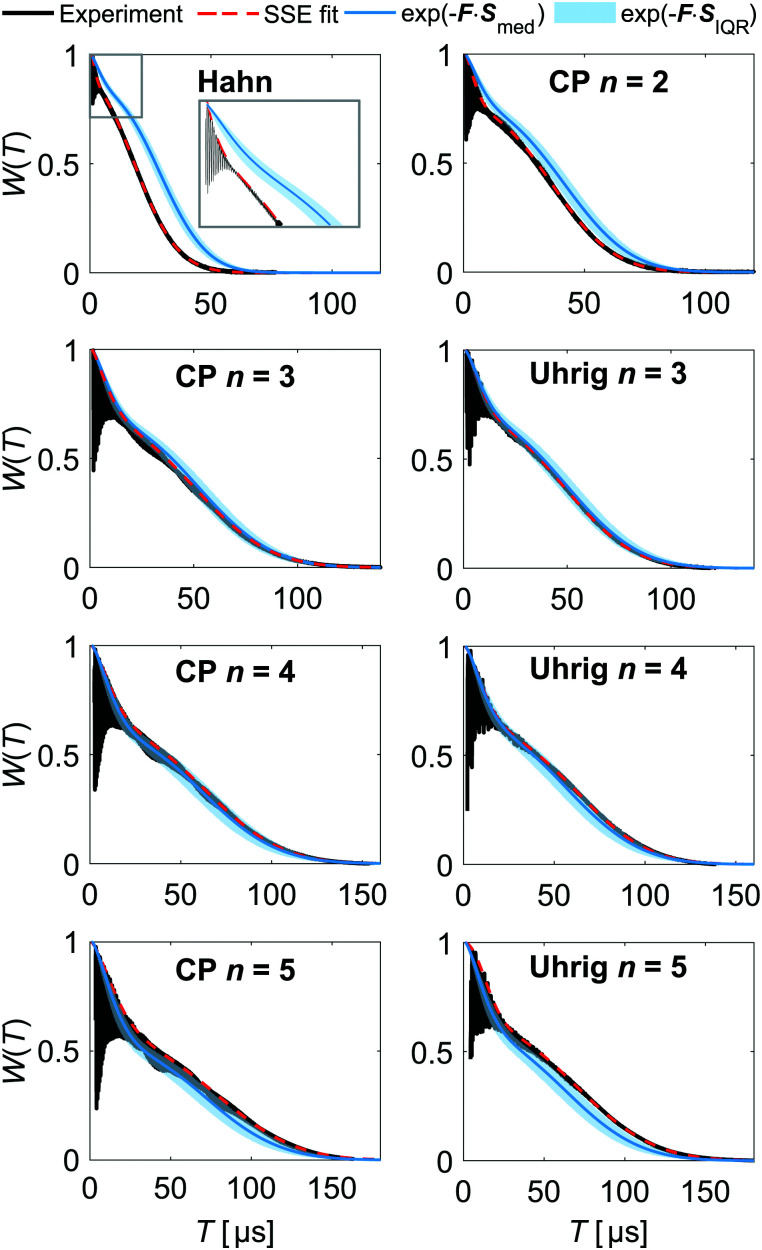
Experimental DD results of 20 μM D-mNOHex in dOTP at 40 K compared to SSE and noise spectrum description. Measured decoherence (black) under Hahn (illustrative inset: the initial vertical variation of the experimental signal stems from nuclear modulation), CP *n* = 2–5 and Uhrig *n* = 3–5 experiment and the associated SSE fit (red, dashed). These parameterizations were used to determine experiment-specific noise spectra *via* regularization. The median ***S***_med_ and interquartile range ***S***_IQR_ of the obtained set of noise spectra (displayed in Fig. 4(a) and (b)) provide a global DDNS-based decoherence description. Using the experiment-specific filter expression **F** generate the associated decoherence function *W*(*T*) (blue) based on ***S***_med_ (blue line) and ***S***_IQR_ (blue shaded area) as specified in the legend on top.

Tests of this constrained regularization procedure with simulated input ***χ*** = −ln(***W***) from known noise spectra have demonstrated that even the smallest level of electronic noise in the decoherence signal ***W*** prohibits the reconstruction of the true underlying noise spectrum (see further details in the ESI,† including Fig. S1). We therefore parameterize the experimental data§§In case nuclear modulations dominate the beginning of the decoherence signal, the parameterization passes through the maxima of the oscillations to correctly describe the underlying decoherence envelope.^15,16^ See the inset of Fig. 3 for an illustration. to achieve a de-noised decoherence description, relying on the stretched exponential (SE) model *V*(*T*) = *c*·exp[−(*T*/*T*_m_)^*ξ*^] with amplitude *c*, and the sum of two stretched exponentials (SSE) model defined by4
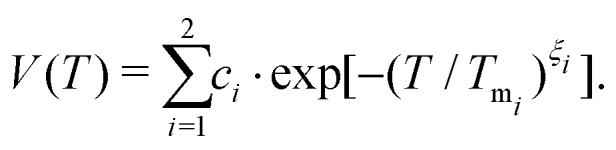


This parameterization captures two separate decoherence pathways associated with a fast (*i* = 1) and a slow (*i* = 2) contribution to the detected echo intensity *V*(*T*), while normalization to *V*(*T* = 0) yields *W*(*T*).

**Fig. 4:**
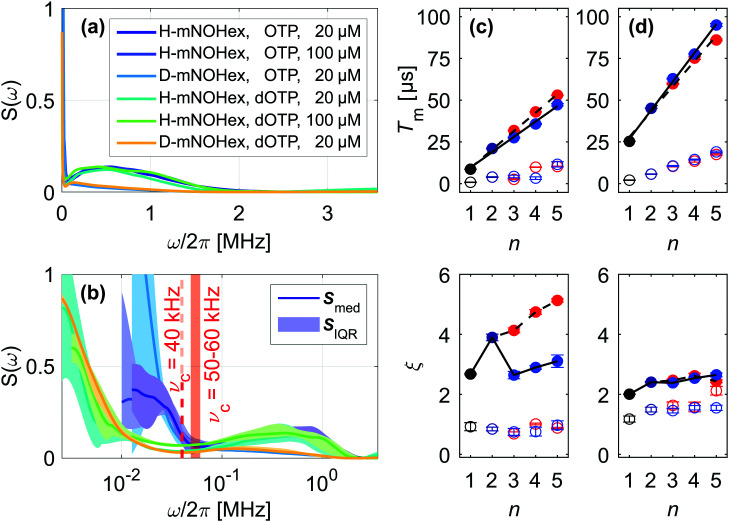
Noise spectra in *o*-terphenyl glass. Median noise spectra ***S***_med_ of H- and D-mNOHex in OTP and dOTP presented on a (a) linear and (b) logarithmic frequency axis with ***S***_med_ (line) and ***S***_IQR_ (shaded area). The cut-off frequency *ν*_c_ is indicated for OTP (red, shaded area) and dOTP (red, dashed line). SSE parameters *T*_m*i*_ and *ξ*_*i*_ as a function of *n* for (c) 20 μM H-mNOHex in OTP and (d) 20 μM D-mNOHex in dOTP. Different DD experiments are color-coded with Hahn (black), CP *n* = 2 (dark blue), CP *n* > 2 (blue) and Uhrig *n* > 2 (red), while filled and empty symbols represent slow (*i* = 2) and fast (*i* = 1) SSE contributions, respectively. The *T*_m_ subplot shows linear regression lines for *T*_m2_ increase with *n* for CP (black, solid) and Uhrig (black, dashed), while lines in the *ξ* subplot guide the eye.

**Fig. 5:**
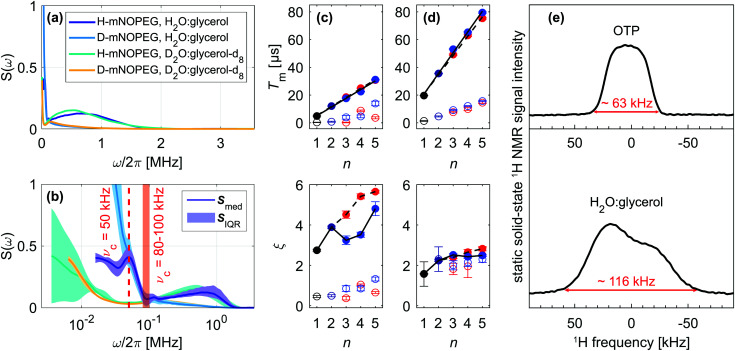
Noise spectra in water–glycerol glass and static ^1^H NMR linewidth. Median noise spectra ***S***_med_ of H- and D-mNOPEG in H_2_O:glycerol and D_2_O:glycerol-d_8_ presented on a (a) linear and (b) logarithmic frequency axis with ***S***_med_ (line) and ***S***_IQR_ (shaded area). The cut-off frequency *ν*_c_ is indicated for H_2_O:glycerol (red, shaded area) and D_2_O:glycerol-d_8_ (red, dashed line). SSE parameters *T*_m*i*_ and *ξ*_*i*_ as a function of *n* for (c) 10 μM H-mNOHPEG in H_2_O:glycerol and (d) 10 μM D-mNOPEG in D_2_O:glycerol-d_8_. Different DD experiments are color-coded with Hahn (black), CP *n* = 2 (dark blue), CP *n* > 2 (blue) and Uhrig *n* > 2 (red), while filled and empty symbols represent slow (*i* = 2) and fast (*i* = 1) SSE contribution, respectively. The *T*_m_ subplot shows linear regression lines for *T*_m2_ increase with *n* for CP (black, solid) and Uhrig (black, dashed), while lines in the *ξ* subplot guide the eye. (e) Static solid-state ^1^H NMR solid echo spectra of OTP and H_2_O:glycerol recorded at 14.1 T and 100 K.

**Fig. 6:**
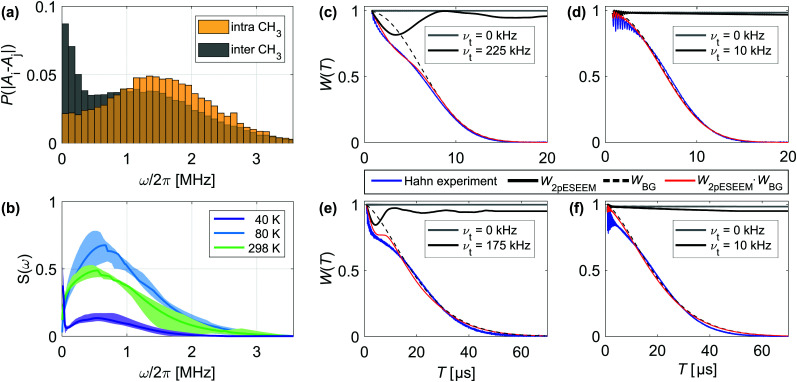
Effect of intramolecular methyl groups on nitroxide coherence. (a) Probability distribution of HF coupling offset |*A*_*i*_ − *A*_*j*_| between two methyl protons *i* and *j* that reside on the same (yellow) or different (gray) methyl group of the nitroxide. (b) Median noise spectra ***S***_med_ (line) and ***S***_IQR_ (shaded area) of 20 μM H-mNOHex in OTP as a function of temperature. (c)–(f) Effect of rotational tunneling on Hahn coherence measurements at 40 K and 20 μM spin concentration (blue). The simulation (red) relies on the solvent-specific background function *W*_BG_, modeled by an SE expression with *c* = 1, (black, dashed) and the *ν*_t_-dependent quantum rotor two-pulse ESEEM *W*_2pESEEM_ with *ν*_t_ = 0 kHz in absence (gray, solid) and presence of rotational tunneling (black, solid). For deuterated methyl groups quadrupole coupling with *ν*_NQ_ = 200 kHz was taken into account. (c) H-mNOHex in OTP with *ν*_t,CH3_ = 225 kHz, (d) D-mNOHex in OTP with *ν*_t,CD3_ = 0–10 kHz and *W*_BG_ parameters *T*_m_ = 8.0 μs and *ξ* = 2.30, (e) H-mNOHex in dOTP with *ν*_t,CH3_ = 175 kHz, (f) D-mNOHex in dOTP with *ν*_t,CD3_ = 0–10 kHz and *W*_BG_ parameters *T*_m_ = 22.8 μs and *ξ* = 1.55.

Given a series of DD measurements, it is advantageous to perform experiment-specific regularized DDNS and determine the statistical median noise spectrum ***S***_med_ and its associated interquartile range (IQR) ***S***_IQR_ from the set of inferred noise spectra. Compared to a global regularization approach, we find that ***S***_med_ achieves a superior agreement with experimental data. In addition, this procedure does not average potential signal variations stemming from either pulse imperfections or non-Gaussian contributions to the noise process. Both of these effects scale with *n*, yet the Uhrig sequence is by design less efficient in suppressing non-Gaussian features compared to CP refocusing.^5^***S***_IQR_ reflects the variability across a DD data set and is thus represented as a colored band surrounding ***S***_med_ (see Fig. 4(b), 5(b) and 6(b)). Combining ***S***_med_ with ***S***_IQR_ thus provides a global decoherence description. Fig. 3 depicts the agreement of this DDNS-based model with experimental DD traces of D-mNOHex in deuterated *o*-terphenyl (dOTP), recorded using the Hahn, CP and Uhrig sequence with *n* ≤ 5 at 40 K. This case is representative (see Fig. S5–S7, ESI† for all other DD traces), as it demonstrates the adequate decoherence descriptions for acquisitions relying on *n* > 1. ***S***_med_ and the associated interquartile range ***S***_IQR_ also capture the shape of the Hahn decay, but they systematically overestimate the associated decoherence time. This observation originates from the filter function bandwidth that narrows with *n* and *T*. Compared to sequences with *n* > 1, the wider Hahn filter allows for a less specific *S*(*ω*) to *W*(*T*) mapping, and *vice versa* (Fig. 2). The Hahn experiment is therefore limited twofold: first, the associated decay does not allow for a reliable noise spectrum reconstruction, unless *W*(*T*) is detectable for *T* ≥ 50 μs. Second, a conventional decoherence analysis that is exclusively based on the Hahn decay may not reveal all contributing dephasing mechanisms.^16^

### 2.2 Noise spectra: low temperature regime (40 K)

We have determined the low-temperature noise spectra of nitroxides in *o*-terphenyl (Fig. 4) and water–glycerol (Fig. 5) glass from DD data sets acquired at 40 K. Independent of the solvent, the noise spectra consist of two separate features, visible on a linear frequency axis (Fig. 4(a) and 5(a)) and better resolved by logarithmic scaling (Fig. 4(b) and 5(b)). The low-frequency contribution extends up to a cut-off frequency *ν*_c_ and originates from matrix nuclei, as demonstrated by solvent per-deuteration. The cut-off frequency is a central concept in the DD^1,4,10,11,14,18,19^ and DDNS^5,8,9^ literature. Broadly speaking this parameter indicates that for *ω* > *ω*_c_*S*(*ω*) → 0,^1^ whereas specific noise spectrum models define the cut-off frequency rigorously *via* a functional expression, *e.g. S*(*ω*) ∝ *ωΘ*(*ω*_c_ − *ω*)^4^ or *S*(*ω*) ∝ *ω*^−2^ exp[−(*ω*/*ω*_c_)^2^].^19^ Given our model-free DDNS approach, we rely on this terminology to refer to the local minimum (marked *ν*_c_ in Fig. 4(b) and 5(b)) of the here determined two component noise spectra, as the cut-off frequency curtails the solvent-induced noise spectral density. Using isotope substitution, we assign the spectral component at higher frequencies, *i.e.* above *ν*_c_, to the protons and deuterons that reside on the two pairs of geminal methyl groups of the nitroxide molecule (Fig. 1(d) and (e)). Naturally, these observations reflect results from our previous studies, as refocusing experiments sample *S*(*ω*) from the highest (*ω*_max_, Fig. S2, ESI†) to lowest (*ω*_min_, Fig. S3, ESI†) accessible frequency by incrementing *T* (Fig. 2). Hence, the two noise spectral features capture the fast, nitroxide-driven and the slow, solvent nuclei-driven decoherence pathways that are observable in the low temperature (10–60 K) by Hahn and DD experiments and required the introduction of the SSE model.^15,16^ Generally, the *n*-dependence of *T*_m_ and *ξ* has prove a powerful representation to capture, characterize and compare the DD effect under various experimental conditions.^11,15,16,19–22^ In the following, we thus link the SSE parameters *T*_m_*i*__ and *ξ*_*i*_ for this fast (*i* = 1) and slow (*i* = 2) contribution to features of the noise spectrum and relate them to active decoherence mechanisms.

DD continuously prolongs the decoherence times *T*_m_1__ and *T*_m_2__ for *n* = 1–5 in all here considered cases, demonstrating the suppression effect of the associated filter functions *F*(*ωT*). We show the resulting scaling of the SSE parameters for the fully protonated spin systems H-mNOHex in OTP (Fig. 4(c)) and H-mNOPEG in H_2_O:glycerol (Fig. 5(c)) and their deuterated analogues D-mNOHex in dOTP (Fig. 4(d)) and D-mNOPEG in D_2_O:glycerol-d_8_ (Fig. 5(d)). The linear increase of *T*_m_2__ with *n* is particularly characteristic and arises from the small cut-off frequency^19^ of *ν*_c_ = 40 to 100 kHz, that curtails the majority of the low-temperature noise spectrum. Mechanistically, this spectral contribution depicts nuclear spin diffusion (NSD), which drives electron spin decoherence up to a correlation time *t*_c_ = 1/*ν*_c_.^19^ In this process, *N* solvent nuclei are coupled to each electron spin, generating a hyperfine (HF) field 
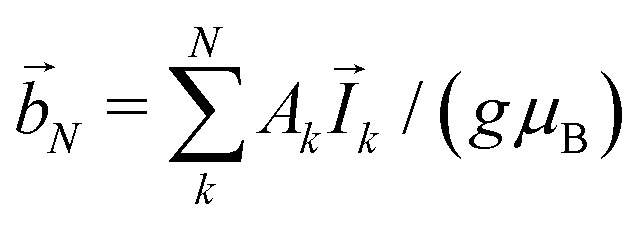
 that fluctuates due to flip-flop transitions of dipolar coupled homonuclear pairs with index *i* and *j*. The difference in their HF interactions |*A*_*i*_ − *A*_*j*_| modulates the longitudinal component *b*_*N*,*z*_ (neglecting *b*_*N*,*x*_, *b*_*N*,*y*_ ≪ *B*_0_), given that the pair's dipolar coupling *d*_*i*,*j*_ compensates the generated energy offset. As a consequence NSD is suppressed for |*A*_*i*_ − *A*_*j*_| > *d*_*i*,*j*_ and the cut-off frequency *ν*_c_ reflects this limit. Experimentally, solvent deuteration shifts this parameter from *ν*_c_ = 50–60 kHz (OTP) to 40 kHz (dOTP) (Fig. 4(b)) by scaling the HF and dipolar interactions, and by introducing quadrupolar couplings that influence the NSD condition. The reduced cut-off frequency in dOTP enables longer decoherence times and a steeper increase of *T*_m_2__ with *n* (Fig. 4(d)) compared to OTP (Fig. 4(c)). While the Uhrig sequence achieves larger *T*_m_2__ and *ξ*_2_ values in OTP (Fig. 4(c)), CP delays are more effective in decoupling deuteron-driven NSD (Fig. 4(d)). This performance difference arises from the respective filter properties: Uhrig delays are optimized to suppress low-frequency contributions of the noise spectrum,^4^ which have larger amplitudes in OTP compared to dOTP (Fig. 4(b)), leading to *ξ*_2_(OTP) > *ξ*_2_(dOTP). Overall, we obtain related results in water–glycerol glass, where deuteration reduces *ν*_c_ of 80–100 kHz in H_2_O:glycerol to 50 kHz in D_2_O:glycerol-d_8_ (Fig. 5(b)). Compared to *o*-terphenyl these cut-off frequencies are larger (*ν*_c_(H_2_O:glycerol) > *ν*_c_(OTP), *ν*_c_(D_2_O:glycerol-d_8_) > *ν*_c_(dOTP)) and explain the following observations. First, the advantage of the Uhrig over CP decoupling in H_2_O:glycerol (Fig. 5(c)) is reduced relative to OTP, and *vice versa* in D_2_O:glycerol-d_8_ (Fig. 5(d)), in line with *S*(*ω*) components at higher frequencies that offset the Uhrig advantage.^19^ Second, in water–glycerol glass we observe decreased *T*_m_2__/*n* slopes and shorter *T*_m_2__ values, rendering the *T*_m_1__ and *T*_m_2__ time scales more comparable. The solvent- and nitroxide feature of the water–glycerol noise spectra are thus spectrally less distinct. Under unfavorable conditions, *e.g.* D-mNOPEG in H_2_O:glycerol, the Hahn decay is unable to resolve the two spectral components^16^ and DD sequences with narrower filter functions are required to identify the contributing decoherence processes.

Under limited refocusing, we also observe *ξ*_2_(*n* = 2) > *ξ*_2_(*n* = 1) for both matrices (Fig. 4(c) and 5(c)). Generally, this dependence of *ξ* on *n* is known as pulse-number parity effect and originates from different orders of many-body nuclear correlations that dominate electron spin decoherence under DD if the associated nuclear correlation time *t*_c_ exceeds *T*_m_.^20,23^ Consequently, for *n* > 2 *ξ*_2_ deviates from the even-odd pattern as DD extends *T*_m_ beyond the nuclear correlation time *t*_c_ = 1/*ν*_c_ of 16–20 μs and 10–12.5 μs for OTP and H_2_O:glycerol, respectively. The same “partial” even-odd effect has also been noted for irradiated crystal malonic acid,^21^ but not interpreted.

Overall, these results show that the cut-off frequency provides an effective description of the coupled spin network that facilitates NSD. Given that the hyperfine interactions to OTP and H_2_O:glycerol protons are comparable, an increase in *ν*_c_ directly reports on a larger dipolar reservoir that drives NSD. On a molecular level, this reflects a reduction of the shortest intermolecular ^1^H-^1^H distances present in H_2_O (∼1.5 Å) and glycerol (∼1.8 Å) relative to OTP (∼2.5 Å). Similarly, for irradiated crystal malonic acid such a proton pair control has been demonstrated as particular orientations of all adjacent proton pairs determine the decoherence behavior.^21^ Low-temperature (100 K) measurements of the static solid-state ^1^H NMR spectra of OTP and H_2_O:glycerol (in absence of the electron spins) (Fig. 5(e) and Fig. S8, ESI†) show broad lines with width of approximately 63 and 116 kHz, respectively, which manifest predominantly the strong nuclear dipole–dipole coupling of protons in the solvents. This demonstrates that the different ^1^H–^1^H distances translate into a more tightly dipolar coupled solvent proton bath for H_2_O:glycerol compared to OTP glass.

Besides NSD, the *n* π pulses with lengths in the nanosecond range can induce instantaneous diffusion (ID) of the pulse-excited electron spins,^24^ as evolution of their dipolar couplings amounts to local field changes *b*_*z*_(*t*). The total electron spin concentration and the pulse excitation bandwidth determine the impact of this mechanism. Usually, ID is weighted against NSD when contributing decoherence processes are discussed, stating that proton-driven NSD masks ID effects, while deuterated systems are sensitive to this mechanism.^15,25,26^ In DDNS terms, we observe a minor increase in the *S*(*ω*) amplitude for 100 μM H-mNOHex in OTP compared to 20 μM (Fig. 4(b)). As the dOTP induced noise power is confined below *ν*_c_ = 40 kHz, the same change in concentration has a larger relative effect on the overall noise spectra, best visible in the frequency range of *ω*/2π = 15–100 kHz (Fig. 4(b)). The spectral feature associated with H-mNOHex conceals ID contributions at somewhat higher frequencies, which are detectable by pulse-length dependent Hahn experiments involving 100 μM D-mNOHex dissolved in OTP and dOTP.^15^

Deuterating the geminal methyl groups of the nitroxide molecules alters the corresponding noise spectrum feature, affecting its position, width and amplitude (Fig. 4(a) and 5(a)). Empirically, nitroxide deuteration slows *T*_m_1__ and leads to a larger *ξ*_1_ value and steeper increase of *T*_m_1__ with *n* compared to its protonated analog (Fig. 4(c), (d) and 5(c), (d)). Any nuclear spin-induced mechanism that drives the associated coherence loss needs to overcome the energy offset |*A*_*i*_ − *A*_*j*_| between two protons or deuterons with index *i* and *j* that reside on the four methyl groups. This quantity can be estimated based on electron-nuclear spin distances *r*_*i*_ obtained from a 0.5 ns molecular dynamics (MD) trajectory of a protonated nitroxide at room temperature (RT). We approximate the hyperfine coupling *A* by the point-dipole electron-nuclear interaction term *a*_*i*_ = *μ*_0_*g*_e_*μ*_B_*γ*_n_/4π*r*_*i*_^3^ with the magnetic constant *μ*_0_, a *g*_e_ value of 2, the Bohr magneton *μ*_B_ and the gyromagnetic ratio *γ*_n_. The resulting |*A*_*i*_ − *A*_*j*_| distribution arises from intra- and inter-methyl group pairs (Fig. 6(a)), while methyl group deuteration scales the width of this distribution by the gyromagnetic ratio *γ*_n_(^1^H)/*γ*_n_(^2^H) = 6.5. The shape of the computed HF offset distribution agrees particularly well with the *S*(*ω*) component assigned to the protonated methyl groups, thus highlighting the importance of this structural entity for the fast coherence process. Similar to solvent nuclei, these methyl protons and deuterons can undergo NSD, given that their nuclear spin–spin interaction compensates |*A*_*i*_ − *A*_*j*_|. The strongest intramolecular proton–proton interaction amounts to ∼35 kHz, which limits the purely dipolar-driven mechanism to a few proton pairs residing on the same methyl group.^27^ Consequently, an additional effect must contribute to generate noise spectral features beyond this dipolar limit.

Low temperatures, such as 40 K, freeze out classical methyl group rotation, as the available thermal energy is not sufficient to overcome the rotation barrier *E*_rot_. Instead, the rotor of *C*_3_ symmetry populates the rotational ground state (*m* = 0 with *E*_*m*=0_) of the three localized substates that correspond to the minima of the associated potential. The height of this potential determines to what extent the localized wave functions overlap to enable coherent rotational tunneling. Recently, the excitation and indirect detection of tunnel coherence has been demonstrated by three-pulse electron spin echo envelope modulation (ESEEM) experiments for two methyl groups of a dimethylammonium cation within a Mn(ii)-doped perovskite framework.^28^ In this case, the determined tunnel frequencies *ν*_t_ match the methyl proton HF couplings in size,^28^ whereas methyl groups bound to a sp^3^ carbon atom typically exhibit smaller *ν*_t_ of 100–300 kHz.^29^ Density functional theory (DFT) calculations of the rotational barrier for one methyl group of the here investigated nitroxide moiety result in a tunnel frequency in this range, as the computed *E*_rot_ = 13.7 kJ mol^−1^ converts into *ν*_t_ = 207 kHz *via* the hindered quantum rotor model.^28,30^ Compared to the HF couplings with the methyl protons, this tunnel frequency is small and only weakly perturbs the HF interactions.^28^ We rely on density operator formalism to simulate the quantum rotor dynamics under the Hahn (also called two-pulse ESEEM) experiment in order to understand whether the tunnel effect explains the observed echo envelope on the *T*_m_1__ time scale. In absence of rotational tunneling, the secular and pseudo-secular HF couplings to methyl protons result in an unmodulated trace at *B*_0_ = 1.224 T, whereas *ν*_t_ = 225 kHz induces a signal with a sizable modulation depth of 0.25 and a period in the microsecond range (Fig. 6(c)). Methyl group deuteration changes the nuclear spin quantum number from *I* = 1/2 to 1, reduces the HF coupling and introduces a rotor-state dependent quadrupole coupling with *ν*_NQ_ = 200 kHz. The latter effect arises from the electric field gradient along the C–D bond and the three different orientations of this bond in a CD_3_ group relative to the outer magnetic field. Moreover, deuteration doubles the rotors moment of inertia, which typically scales *ν*_t_ by a factor of ∼20 compared to a CH_3_ group, thus strongly attenuating the tunneling effect.^29^ We determine *ν*_t,CH_3__ and *ν*_t,CD_3__ by simultaneously fitting the normalized Hahn decays of a protonated nitroxide and its deuterated analogue in the same solvent by the products *W*_2pESEEM,CH_3__·*W*_BG_ and *W*_2pESEEM,CD_3__·*W*_BG_, respectively. This approach accounts for two independent signal contributions: first, a background decay *W*_BG_ that we model by an SE expression and as discussed mainly reflects the solvent-induced decoherence process. Second, the modulation of the Hahn echo *W*_2pESEEM_ that arises from partial excitation of the tunnel transition by the π/2 pulse and displays a *ν*_t_-dependent modulation depth and frequency (Fig. S9, ESI†). We rely on a set of pre-simulated *W*_2pESEEM,CH_3__ and *W*_2pESEEM,CD_3__ for a range of *ν*_t,CH_3__ and *ν*_t,CD_3__ values to extract *ν*_t,CH_3__ = 175–225 kHz (Fig. 6(c) and (e)) and *ν*_t,CD_3__ = 0–10 kHz (Fig. 6(d) and (f)) from fits to Hahn decays of H- and D-mNOHex in OTP and dOTP. Naturally, a protonated solvent attenuates *W*_2pESEEM_ more strongly than its deuterated version, thus explaining the somewhat larger tunnel frequency obtained from Hahn data in OTP (*ν*_t,CH_3__ = 225 kHz, Fig. 6(c)) compared to dOTP (*ν*_t,CH_3__ = 175 kHz, Fig. 6(e)). Analogously, we determine *ν*_t,CH_3__ = 275 kHz and 225 kHz in H_2_O:glycerol (Fig. S10(a), ESI†) and D_2_O:glycerol-d_8_ (Fig. S10(b), ESI†), respectively, given the faster background decay in water–glycerol compared to *o*-terphenyl glass. Overall, the extracted *ν*_t,CH_3__ values match the DFT-based prediction in case of the protonated nitroxide and strongly suggest that rotational tunneling drives the methyl proton-induced *S*(*ω*) contribution. Instead, it is challenging to determine *ν*_t,CD_3__ accurately. On the one hand, because of *ν*_t,CD_3__ ≪ *ν*_t,CH_3__ so that the *ν*_t,CD_3__ candidates generate only small differences in modulation depth (Fig. S9(d), ESI†). On the other hand, because forbidden electron-deuteron transitions contribute to *W*_2pESEEM,CD_3__. While we cannot state with certainty whether rotational tunneling is fully suppressed in D-mNOHex and D-mNOPEG, it is clear that methyl group deuteration strongly dampens the tunneling effect for *gem*-dimethyl nitroxides.

Beyond the above-discussed results obtained for the Hahn/two-pulse ESEEM sequence, DD experiments with *n* > 1 improve the detection of the rotational tunneling contributions twofold. In addition to the background decay, the refocusing pulses also affect the CH_3_ tunnel decoherence *W*_2pESEEM,CH_3__ as evident from simulated CP *n* = 2–4 ESEEM signals (Fig. S10, ESI†). Compared to the excellent fit quality of *W*_2pESEEM,CH_3__·*W*_BG_ to Hahn traces of H-mNOPEG in H_2_O:glycerol (Fig. S10(a), ESI†) and D_2_O:glycerol-d_8_ (Fig. S10(b). ESI†), the agreement between the corresponding products *W*_ESEEM,CH_3__·*W*_BG_ for CP *n* = 2–4 with experimental data is only reasonable. The observed deviation is likely to arise from a distribution of tunnel frequencies, expected for the glassy state, to which DD experiments with *n* > 1 appear to be more sensitive compared to the Hahn sequence.

### 2.3 Noise spectra: intermediate (80 K) to high temperature (298 K)

Raising the temperature above the low-temperature limit strongly reduces the decoupling efficiency for *gem*-dimethyl nitroxides, as previously demonstrated for DD measurements in *o*-terphenyl at 80 and 298 K^15^ (Fig. S6b, ESI†). This observation is mirrored by the corresponding noise spectra of 20 μM H-mNOHex in OTP that lack a cut-off frequency to confine the noise power as observed at 40 K (Fig. 6(b)). Specifically, the noise spectra at 80 and 298 K consist of a single feature, the methyl group-induced *S*(*ω*) component, with a temperature-dependent amplitude. Evidently, the same |*A*_*i*_ − *A*_*j*_| distribution that contributes to the DD traces at 40 K, induces dephasing at higher temperature, while the driving mechanism transitions from quantum to classical methyl group dynamics (Fig. 6(b)). At low temperatures, the relatively small tunnel frequency (*ν*_t_ < HF coupling) limits the methyl-group induced effect as reflected by the comparably small amplitude of the corresponding *S*(*ω*) feature. In contrast, as a thermally activated process the reorientation rate *τ*_c_^−1^ of the classical methyl group dynamics is temperature-dependent. At a certain temperature, *τ*_c_^−1^ matches |*A*_*i*_ − *A*_*j*_| in size, so that the classical methyl group rotations modulate the electron spin most effectively and produces a minimal *T*_m_. For H-mNOHex in OTP this condition is fulfilled at 140 K as the dephasing time of the temperature-dependent Hahn echo decay decreases from the low-temperature limit to 140 K and increases again above 140 K.^15^ Therefore, the larger *S*(*ω*) amplitude observed at 80 K originates from a reorientation rate *τ*_c_^−1^ that matches |*A*_*i*_ − *A*_*j*_| more closely than at 298 K (Fig. 6(b)), while *τ*_c_^−1^ < |*A*_*i*_ − *A*_*j*_| and *τ*_c_^−1^ > |*A*_*i*_ − *A*_*j*_| applies at the two temperature points, respectively.

## 3 Conclusions

Regularized DDNS lifts the limitation imposed by the *δ*-function approximation central to the common noise spectroscopy formalism. Similar to a recent deep-learning DDNS implementation that can deal with experimental noise directly,^31^ our regularized approach makes DDNS thus applicable to DD measurements recorded with few refocusing pulses. Thereby, it enables noise spectrum reconstruction for spin systems that do not qualify for qubit candidates, but exhibit sufficient long dephasing times to allow for multiple refocusing. Amongst others, organic radicals in glassy matrices fulfill these low requirements. Applying regularized DDNS to nitroxides in *o*-terphenyl and water–glycerol glass, we demonstrate that the obtained noise spectra provide a tangible decoherence description even in the disordered glassy state: firstly, a noise spectrum visualizes the fluctuating spin environment that induces dephasing. It is thus more comprehensive than *T*_m_ and *ξ* values obtained from a conventional Hahn decay analysis. Secondly, it reflects variation in temperature, deuteration, paramagnetic species and spin concentration, facilitating assignment of contributing noise spectrum features. Thirdly, given their quantitative nature, the noise spectra reveal mechanistic details regarding the fast, nitroxide-induced process and the slow, solvent-induced NSD, in the form of a characteristic cut-off frequency *ν*_c_. Solvent deuteration reduces *ν*_c_ and translates into a superior decoupling effect by the CP compared to the Uhrig sequence. Usually, such a performance difference is discussed in terms of a “soft” rather than “hard” cut-off, referring to a noise spectrum that levels off slowly instead of abruptly beyond *ν*_c_.^5,6,18,19^ While these two *S*(*ω*) characteristics (size of *ν*_c_ and behavior beyond *ν*_c_) are similar in describing the noise power distribution, they do differ, indicating that it is dangerous to infer the *S*(*ω*) shape from only indirect information, such as relative performance of CP and Uhrig DD.

By means of density operator formalism, we furthermore confirmed that rotational tunneling of the methyl groups drives the fast decoherence process observed in the Hahn decay of H-mNOHex and H-mNOPEG. We therefore provide experimental evidence for the two-pulse tunnel ESEEM, predicted already in 1998.^32^ Our data and simulations furthermore suggest that DD experiments are superior in monitoring this tunnel effects from strongly hindered CH_3_ groups (small *ν*_t_) compared to the Hahn/two-pulse ESEEM experiment. On the one hand, this may explain why, despite decades of nitroxide relaxation studies, this contribution has been overlooked. On the other hand, DD offers an experimental handle to investigate methyl tunnel states including deuteration, matrix and rotor-rotor coupling effects in the future more closely. Our DFT-based estimates of the rotational coupling strength for the here studied *gem*-dimethyl nitroxide moiety in vacuum suggest a non-negligible contribution, while further investigations are needed to clarify its importance in the solvated state.

A recent study recognized the contribution from rotational tunneling for organic polycrystals featuring a methyl group and implemented this term for cluster correlation expansion (CCE) calculations.^22^ Compared to simulations in absence of tunneling, the simulated Hahn decays result in shortened or prolonged matrix-induced dephasing times for small (*ν*_t_ ∼ 100 kHz) or large (*ν*_t_ ∼ 5 MHz) tunnel frequencies, respectively.^22^ The former case matches observations of an earlier experimental study based on Hahn traces recorded at 40 K for TEMPONE in glassy solvents that featured strongly hindered methyl groups.^25^ The CCE-based study also includes simulated CP *n* ≤ 6 traces that predict a decoupling effect for both small and large tunnel frequencies.^22^ Therefore, future DDNS studies should investigate solvents that contain either strongly or weakly hindered methyl groups. We anticipate that the resulting noise spectra will reflect the *ν*_t_ and thus *E*_rot_ distribution expected for the glassy state, obtaining valuable information about the underlying spin dynamics.

Beyond this fundamental interest to study unbound radical molecules, regularized DDNS may serve as a biophysical tool itself: investigations of spin-labeled biomolecules can characterize other dynamic processes, such as phenyl ring flips, known to occur in proteins containing phenylalanine and tyrosine. Furthermore, detecting the HF field induced by the biomolecules backbone against a deuterated solvent may provide coarse-grain information on the biomolecules conformation.

In this work, we have utilized noise spectra as decoherence descriptors. Inversely, they can also serve as decoherence predictors. Having a sample-specific noise spectrum in hand allows for *in silico* optimization of pulsed EPR experiments, in particular refocusing schemes. Thereby distance measurements by dipolar spectroscopy can access longer distances after adjusting the observer sequence of the 4-pulse,^26^ 5-pulse,^33,34^ 7-pulse^35^ and *n*-DEER^36^ experiments accordingly. Regularized DDNS can potentially benefit from new pulse sequences, if optimized filter functions sample the underlying noise spectrum more effectively, improving noise spectrum reconstruction compared to the established CP and Uhrig sequences.

Ultimately, the first implementation of noise spectroscopy for a superconducting qubit^6^ has led to a rich body of research for qubit systems.^9^ We have demonstrated that the noise spectra of nitroxides in glassy matrices provide fundamental insights into an incomparably more complex spin environment. In light of these results, we envision that future applications of regularized DDNS to spin systems for basic, biological and chemical research will generate a better understanding of the decoherence-driving spin dynamics that can in turn lead to improvements in sensitivity and resolution.

## 4 Materials and methods

### 4.1 Sample preparation

Four different *gem*-dimethyl mono-nitroxide (mNO) radicals were investigated, featuring either a protonated nitroxide moiety (Fig. 1(d)) or its per-deuterated analogue (Fig. 1(e)). We previously reported the synthesis of H-mNOHex and its partially deuterated variation D-mNOHex.^15^ We also published the synthesis of the water-soluble nitroxides H-mNOPEG and D-mNOPEG,^16^ and characterized its self-aggregation behavior at concentrations >30 μM. H-mNOHex and D-mNOHex were dissolved in either OTP or dOTP to spin concentrations of 20 and/or 100 μM. To prevent aggregate formation, H-mNOPEG and D-mNOPEG were diluted in equivolume mixtures of water and glycerol to a spin concentration of 10 μM, using either H_2_O:glycerol or D_2_O:glycerol-d_8_. The resulting mixtures were filled into 3 mm o.d. quartz capillaries to a filling height of 80 mm without degassing. Samples containing *o*-terphenyl were melted with a heat gun at 70 °C under normal atmosphere prior to shock-freezing in liquid nitrogen, while samples with water–glycerol mixtures were directly immersed into the cryogenic liquid. Between measurements, *o*-terphenyl samples were stored in the fridge at 4 °C, while the glassy frozen state of all water–glycerol samples was maintained by storing in liquid nitrogen. OTP was purchased from ABCR, glycerol from Acros Organics, and D_2_O as well as glycerol-d_8_ from Sigma-Aldrich.

### 4.2 EPR measurements

All electron spin coherence measurements were carried at Q-band frequencies (∼34.35 GHz) at the maximum of the nitroxide spectrum (*B*_0_ ∼ 1.220 T), using a Bruker ElexSysII E580 spectrometer equipped with a 200 W traveling wave tube amplifier and a home-built resonator accepting 3 mm o.d. capillaries.^37^ We adjusted the experimental shot repetition time (SRT) depending on the chosen temperature, thereby preventing that *T*_1_ effects saturate the Hahn echo intensity. DD experiments were performed with constant-bandwidth rectangular pulses of *t*_π_ = *t*_π/2_ = 12 ns length. The number of π pulses *n* determines the complexity of the full phase cycle to detect the correct coherence pathway.^15^ Briefly, the first π/2 of all DD pulse sequences is subject to a two-step phase cycle [+(+*x*) − (−*x*)] to remove the receiver offset, while the last π pulse has a constant phase. Thus, a two-step phase cycle is sufficient for the Hahn experiment (*n* = 1, Fig. 1(a)). Sequences with *n* > 1 require a phase cycle of 4^*n*−1^ steps, consisting of nested [+(+*x*) − (+*y*) + (−*x*) − (−*y*)] cycles for all π pulses except the last two, combined with a [+(+*x*) + (−*x*)] cycle for the π pulse with index *n* − 1. Whereas (+*x*),(+*y*),(−*x*) and (−*y*) specify the pulse phase, the additional plus or minus sign determines how the echo intensity of each phase cycle step combines to form the overall signal as detailed in Table S1, ESI.† Please note that we are here correcting phase cycle elements, which we previously misreported.^15,16^

The decoherence decay under two different DD schemes, namely Carr–Purcell (CP) and Uhrig decoupling, was determined by incrementing the total sequence length *T* and integrating the last refocused echo generated by *n* = 1–5 π pulses. For *n* ≤ 2 the CP and Uhrig sequence share the same *n* + 1 interpulse delays Δ*j*. Specifically, a CP scheme relies on *Δ*_1_ = *Δ*_*n*+1_ = *T*/(2*n*) and *Δ*_*j*_ = *T*/*n* for 2 ≤ *j* ≤ *n*, while Uhrig^4^ derived the following optimized delays5
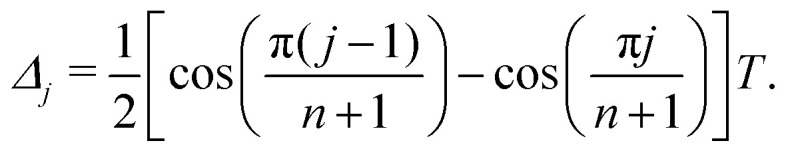
Fig. 1(b) and (c) illustrate the difference in pulse timings for the CP and Uhrig experiment with *n* = 5, respectively.

### 4.3 NMR measurements

The solid-state static ^1^H nuclear magnetic resonance (NMR) measurements of H_2_O:glycerol and OTP were acquired on a Bruker Avance III 600 MHz (14.1 T) DNP NMR spectrometer equipped with low-temperature 3.2 mm double-resonance MAS cryo-probe. The spectra were acquired at 100 K using a solid echo π/2 − *τ* − π/2 −*τ* sequence with an interpulse delay *τ* of 13.3 μs and a 2 μs (125 kHz) ^1^H π/2 pulse. The ^1^H *T*_1_ relaxation times for each sample were measured by saturation recovery and were found to be 38 s and 26 s for H_2_O:glycerol and OTP, respectively. The interpulse delay was correspondingly set to *ca.* 1.4 times the measured *T*_1_ relaxation time. The spectra were background corrected by subtraction of the background spectrum acquired under the same conditions, normalized to the number of scans (Fig. S8, ESI†).

### 4.4 Regularized noise spectroscopy

The DDNS calculations were run with a home-written MATLAB (The MathWorks Inc., Natick, MA) script, relying on the following DeerLab (0.9.2)^38^ functions to implement the regularization approach: regoperator generates second-order discrete differential operator matrices *L*_2_, selregparam chooses the optimal regularization parameter *α* based on the AIC criterion, and to solve eqn (3) a fast non-negative least-square algorithm, implemented in the fnnls function, was used. The central element of DDNS is the filter kernel matrix **F** with elements *F*_*i*,*j*_ = *F*(*ω*_*j*_*T*_*i*_)Δ*ω*/π*ω*_*j*_^2^, where *F*(*ωT*)^5^ refers to the frequency-domain filter function. Eqn (6)–(9) list the *F*(*ωT*) expressions for the here employed DD experiments, namely the Hahn (eqn (6)), Uhrig (eqn (7)) and the CP sequence with an even (eqn (8)) or odd (eqn (9)) number of pulses.6
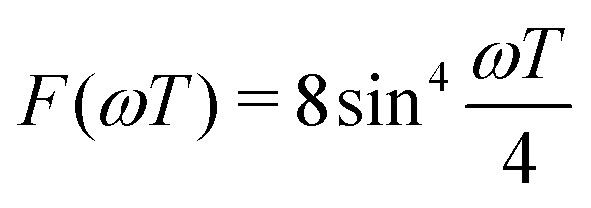
7
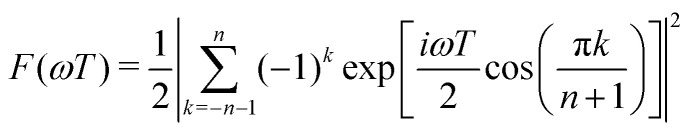
8
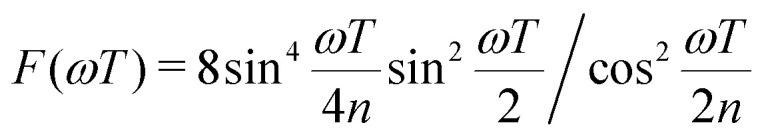
9
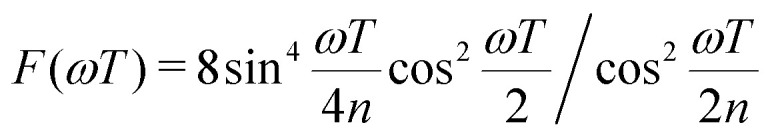


Given an experimental decoherence vector *W*′, the resonator dead-time dictates the first detectable element 
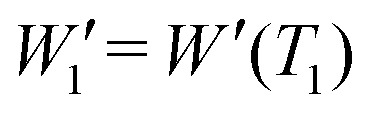
, while the decoherence time of the observed spin system defines the last detectable element 
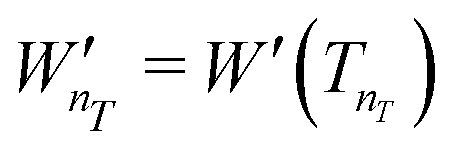
. The corresponding accessible frequency range (*ω*_min_–*ω*_max_) of the noise spectrum is thus determined through the kernel matrix elements *F*_1,*j*_ and *F*_*nT*,*j*_, and we rely on a Gaussian fit (with mean *μ* and standard deviation *σ*) to their main band pass feature to set *ω*_max_ = *μ* + 3*σ* (Fig. S2, ESI†) and *ω*_min_ = *μ* − 3*σ* (Fig. S3, ESI†), respectively. For a DD set with comparable decoherence times (applies to data acquired at 80 and 298 K), DDNS can be performed using a common frequency axis. Noise spectrum reconstruction from Hahn and CP *n* = 2 data typically fails if decoherence times vary with *n* (applies to data acquired at 40 K), while the use of *n*-specific frequency axes mostly solves this problem. We obtain a global noise spectrum ***S***_med_ and the associated interquartile range ***S***_IQR_ by computing the median and IQR from the set of experiment-specific ***S***. As this set is not normally distributed and occasionally features outliers, the median and IQR provide a more robust statistical description compared to the mean and standard deviation. Solutions that fail to model the corresponding decoherence vector ***W*** were excluded, which applies to ***S***_Hahn_ in case of a protonated solvent. Some discontinuities occur in ***S***_med_ and ***S***_IQR_ when computing these quantities from a ***S*** set with *n*-specific frequency axes. By applying a Savitzky–Golay second-order polynomial filter across 201 elements at a time these features are smoothed without substantially affecting the time-domain modeling.

The choice of dimensions *n*_*T*_ and *n*_*ω*_ was optimized by comparing regularized DDNS results for a series of increments Δ*T* = 0.25, 0.125 and 0.0625 μs, while varying the *ω* resolution with 300, 400, 500 or 600 points per 10 MHz of *ω*_max_–*ω*_min_. All presented noise spectra were obtained using 500 points per 10 MHz of *ω*_max_–*ω*_min_, while the optimal time increment Δ*T* varies from 0.25 μs for deuterated solvent, 0.125 and 0.0625 μs for protonated solvents at low and intermediate/high temperature, respectively. Given a linear distribution of *n*_*ω*_ points between *ω*_min_ and *ω*_max_, the multiplication of **F**·***S*** in eqn (3) corresponds to frequency space-integration with a fixed increment Δ*ω*. We find that a logarithmically spaced *ω* vector combined with a vectorized trapezoidal integration allows for a more stable noise spectrum reconstruction by reducing oscillations present in the ***S***_fit_, so that this approach is used throughout. For noise spectra determined from DD data acquired at intermediate (80 K) and high (298 K) temperatures the obtained *S*_fit_ from a log(*ω*) and linear *ω* integration deviates (Fig. S4, ESI†). The width of these noise spectra are thus associated with some uncertainty so that we refrain from interpretation.

### 4.5 MD simulations to sample methyl group conformational space

MD simulations of a protonated nitroxide solved with 5000 H_2_O molecules in a cubic box of 53.4 Å × 53.4 53.4 Å × 53.4 Å were performed using the Material Studio software package (BIOVIA, San Diego, CA, USA). As the used COMPASS II force field lacks the parameterization of the N–O˙ group, we exchanged it for a carbonyl group. First, the system was equilibrated for 0.1 ns at constant *NVT*, followed by 0.1 ns at constant *NPT*. Second, a 0.5 ns MD trajectory (at constant *NVT*) was sampled in 0.5 ps steps and the Cartesian coordinates of the methyl group protons and carbonyl atoms were exported. We approximate the electron density of the N–O˙ group by the midpoint of the carbonyl group to calculate the electron-nuclear spin distance *r*. Using this parameter, the HF coupling to the nitroxides methyl group nuclei was determined, serving as input for the HF offset estimation (Fig. 6(a)).

### 4.6 DFT calculations for tunnel frequency predictions

DFT calculations in ORCA^39^ were used to determine the rotational barrier of the geminal methyl group present in the nitroxide structure in vacuum. After geometry optimization of the simplified version of the here investigated nitroxides (corresponding to Fig. 1(d) with *R*_H_ = H), the rotation barrier *E*_rot_ was determined from a potential energy scan by rotating one methyl group using the B3LYP functional. DFT calculations involving simultaneous rotation of two methyl groups bound to the same carbon atom allow estimation of rotational coupling effects. Based on the free quantum rotor model^30^ implemented in MATLAB as previously described,^28^ we convert *E*_rot_ into a tunnel frequency *ν*_t_.

### 4.7 Tunnel ESEEM simulations: 2pESEEM/Hahn sequence

We simulate the quantum rotor effect under the Hahn experiment by density operator formalism and use a previously reported MATLAB implementation for the three-pulse ESEEM sequence,^28^ adopted to the two-pulse ESEEM, *i.e.* the Hahn experiment with pulse lengths *t*_π_ = *t*_π/2_ = 12 ns. Briefly, the spin Hamiltonian for the three rotational methyl group states depends on the torsional angle *φ*. The high-field approximation applies to the electron spin at *B*_0_ = 1.224 T (orientated along the *z* direction) and we write ℋ_*φ*_ in the electron spin rotating frame for *φ* = 0°10
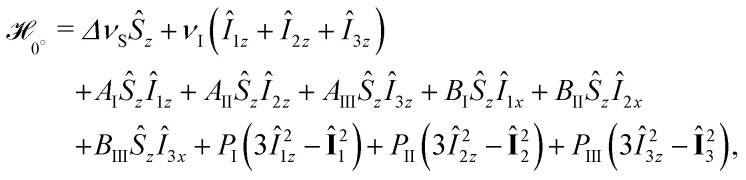
with the electron spin resonance offset Δ*ν*_S_ (we assume Δ*ν*_S_ = 0), the nuclear Zeeman frequency *ν*_I_, the secular *A*_*i*_ and the pseudo-secular *B*_*i*_ hyperfine couplings. For the deuterated nitroxide, the nuclear quadrupole coupling *P*_*i*_ contributes, while we neglect the small asymmetry typical for C–D bonds.^40^ Cyclic permutation of index *i* (I → II → III) generates the spin Hamiltonians for *φ* = 120° and −120°. Using the Easyspin^41^ function sphgrid, we average over the orientation-dependence of the HF couplings *A*_*i*_ = (3 cos^2^ *θ* − 1)*a*_*i*_ and *B*_*i*_ = 3cos *θ* sin *θa*_*i*_ as well as the NQ coupling *P*_*i*_ = (3 cos^2^ *ϕ* − 1)*K* with 
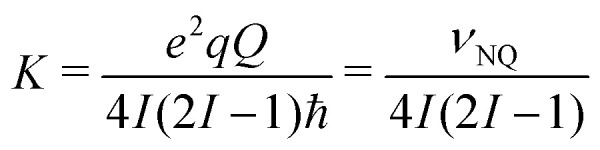
, where *θ* and *ϕ* are the angles between the electron-nuclear spin vector and the C–D bond with the magnetic field direction, respectively. The tunnel effect generates an overlap *ν*_t_/3 between states described by different torsional angle *φ* but identical spin state defined by *m*_S_,*m*_I1_,*m*_I2_,*m*_I3_. The two-pulse ESEEM simulations *V*_2pESEEM_ were performed for the four methyl groups present in the nitroxide structure based on the experimental crystal structure of MTSSL (*S*-(2,2,5,5-tetramethyl-1-oxyl-Δ3-pyrrolin-3-ylmethyl) methanethiosulfonate; CSD-710750)^42^ featuring the same nitroxide moiety as H-mNOHex and H-mNOPEG (*i.e.* corresponding to Fig. 1(d) with *R*_H_ = CH_2_–S–SO_2_CH_3_). The four *V*_2pESEEM_ traces were added up and normalized to obtain *W*_2pESEEM_ (Fig. S9, ESI†). We fit pairs of normalized Hahn traces recorded for a protonated nitroxide and its deuterated analog in the same solvent with the product *W*_2pESEEM,CH_3__·*W*_BG_ and *W*_2pESEEM,CD_3__·*W*_BG_, respectively to determine the tunnel frequencies *ν*_t,CH_3__ and *ν*_t,CD_3__. The solvent-induced background function *W*_BG_ is modeled by an SE expression with *c* = 1, and best fits are determined from pre-simulated *W*_2pESEEM,CH_3__ with *ν*_t,CH_3__ = 150–400 kHz in steps of 25 kHz (selection shown in Fig. S9(c), ESI†) and *W*_2pESEEM,CD_3__ with *ν*_t,CD_3__ = 0, 5, 10, 15, 20, 25, 30, 40, 50, 60 kHz and *ν*_NQ_ = 200 kHz (selection shown in Fig. S9(d), ESI†).

Note that simulations of *W*_2pESEEM,CD_3__ are additionally modulated by forbidden electron-deuteron transitions, which match the experimentally observed fast oscillations at the beginning Hahn trace (*e.g.* well visible in Fig. 6(d) for D-mNOHex in OTP) in frequency but not in modulation depth. The latter originates from correlations of nuclear frequencies of all twelve methyl deuterons present in the nitroxide. These are not included in our simulation of *W*_2pESEEM,CD_3__ as only the slower *ν*_t_-induced modulation of the echo envelope is of interest to explain the fast SSE contribution and CD_3_-induced *S*(*ω*) component.

### 4.8 Tunnel ESEEM simulations: CP *n* = 2–4 sequence

CH_3_-Induced tunnel modulation under DD experiments was simulated by extending the above-described implementation of the Hahn to CP *n* = 2–4 experiments including the associated phase cycles. The larger spin space (2·3·3·3 = 54) faced in case of deuterated methyl groups (compared to 2·2·2·2 = 16 for protonated methyl groups) prohibited corresponding simulations for *n* > 1. Pre-simulated *W*_ESEEM_ under the CP *n* = 2–4 experiments for *ν*_t_ = 150–375 kHz in steps of 25 kHz were used to fit experimental CP *n* = 2–4 traces with the product *W*_ESEEM_·*W*_BG_. Here, *W*_BG_ refers to the experiment-specific background function of a given DD experiment as illustrated for H-mNOPEG in H_2_O:glycerol (Fig. S10(a), ESI†) and D_2_O:glycerol-d_8_ (Fig. S10(b), ESI†).

## Author contributions

Conceptualization: JS, GJ. Methodology: JS. EPR experiments and data analysis: JS. NMR experiments: ZB. Visualization: JS. Software: JS, LFI, GJ. Supervision: YP, GJ. Writing – original draft: JS. Writing – review and editing: JS, LFI, ZB, YP, GJ.

## Conflicts of interest

There are no conflicts to declare.

## Supplementary Material

CP-023-D1CP03103A-s001
