# Supplementary material for: Regularized dynamical decoupling noise spectroscopy – a decoherence descriptor for radicals in glassy matrices
Source: Phys Chem Chem Phys. 2021 Sep 23;23(38):21664–76. doi: 10.1039/d1cp03103a (PMC8494271; doi:10.1039/d1cp03103a)
Supplement: CP-023-D1CP03103A-s001 [file CP-023-D1CP03103A-s001.pdf]

Electronic Supplementary Material (ESI) for Physical Chemistry Chemical Physics.
This journal is © the Owner Societies 2021

# Regularized dynamical decoupling noise spectroscopy – a decoherence descriptor for radicals in glassy matrices

## Supplementary Information

Janne Soetbeer, Luis Fábregas Ibáñez, Zachariah Berkson, Yevhen Polyhach, Gunnar Jeschke

## Table of Contents


1 Phase cycle for dynamical decoupling measurements .......... 1

2 Regularized noise spectroscopy .......... 2

2.1 Impact of electronic noise .......... 2

2.2 Determining the accessible frequency range .......... 3

.......... 6

2.3 DDNS-based decoherence description .......... 6

3 Static solid-state $^1$H NMR spectra .......... 10

4 Tunnel ESEEM simulations .......... 11

4.1 2pESEEM/Hahn sequence .......... 11

4.2 CP *n* = 2-4 sequence .......... 12

5 References .......... 12


## 1 Phase cycle for dynamical decoupling measurements

Table S1 represents the phase cycles employed for the dynamical decoupling (DD) measurements[1,2] as briefly described in Section 4.2 of the main text. The phase cycles of the individual pulses are nested and the order runs from left to right, meaning that the phase of the first pulse is cycled first. Table S1 relies on a *x*, (*x*) and [*x*] notation, which specifies that a pulse is subject to a 360°, 180° and 90° cycle, respectively. This corresponds to the [(+*x*)], [(+*x*) (-*x*)] and [(+*x*) (+*y*) (-*x*) (-*y*)] representation in the main text. Finally, the detection coefficient in Table S1 indicates whether the individual echo intensity is added (+1) or subtracted (-1) to generate the overall signal. This coefficient can be determined for each phase cycle element by multiplying the sign of the employed pulse phase as specified in the main text, *i.e.* [+(+*x*)-(-*x*)] for the first π/2 pulse, [+(+*x*)-(+*y*)+(-*x*)-(-*y*)] and for the penultimate pulse [+(+*x*)+(-*x*)].

**Table S1 Short-hand representation of the DD phase cycle.** *x*, (*x*) and [*x*] indicate that a pulse is cycled in steps of 360°, 180° and 90°, respectively. The individual phase cycles are nested from left to right and the detection coefficient determines whether each element is added (+1) or subtracted (-1).

| **Experiment** | **Pulse phases** | **Detection coefficient** |
|---|---|---|
| $n = 1$ | (*x*)*x* | +1, -1 |
| $n = 2$ | (*x*)(*x*)*x* | +1, -1, +1, -1 |
| $n = 3$ | (*x*)[*x*](*x*)*x* | +1, -1, -1, +1, +1, -1, -1, +1, +1, -1, -1, +1, +1, -1, -1, +1 |
| $n = 4$ | (*x*)[*x*][*x*](*x*)*x* | +1, -1, -1, +1, +1, -1, -1, +1, -1, +1, +1, -1, -1, +1, +1, -1,<br>+1, -1, -1, +1, +1, -1, -1, +1, -1, +1, +1, -1, -1, +1, +1, -1,<br>+1, -1, -1, +1, +1, -1, -1, +1, -1, +1, +1, -1, -1, +1, +1, -1,<br>+1, -1, -1, +1, +1, -1, -1, +1, -1, +1, +1, -1, -1, +1, +1, -1 |
| $n = 5$ | (*x*)[*x*][*x*][*x*](*x*)*x* | +1, -1, -1, +1, +1, -1, -1, +1, -1, +1, +1, -1, -1, +1, +1, -1,<br>+1, -1, -1, +1, +1, -1, -1, +1, -1, +1, +1, -1, -1, +1, +1, -1,<br>-1, +1, +1, -1, -1, +1, +1, -1, +1, -1, -1, +1, +1, -1, -1, +1,<br>-1, +1, +1, -1, -1, +1, +1, -1, +1, -1, -1, +1, +1, -1, -1, +1,<br>+1, -1, -1, +1, +1, -1, -1, +1, -1, +1, +1, -1, -1, +1, +1, -1,<br>+1, -1, -1, +1, +1, -1, -1, +1, -1, +1, +1, -1, -1, +1, +1, -1,<br>-1, +1, +1, -1, -1, +1, +1, -1, +1, -1, -1, +1, +1, -1, -1, +1,<br>-1, +1, +1, -1, -1, +1, +1, -1, +1, -1, -1, +1, +1, -1, -1, +1,<br>+1, -1, -1, +1, +1, -1, -1, +1, -1, +1, +1, -1, -1, +1, +1, -1,<br>+1, -1, -1, +1, +1, -1, -1, +1, -1, +1, +1, -1, -1, +1, +1, -1,<br>-1, +1, +1, -1, -1, +1, +1, -1, +1, -1, -1, +1, +1, -1, -1, +1,<br>-1, +1, +1, -1, -1, +1, +1, -1, +1, -1, -1, +1, +1, -1, -1, +1,<br>+1, -1, -1, +1, +1, -1, -1, +1, -1, +1, +1, -1, -1, +1, +1, -1,<br>+1, -1, -1, +1, +1, -1, -1, +1, -1, +1, +1, -1, -1, +1, +1, -1,<br>-1, +1, +1, -1, -1, +1, +1, -1, +1, -1, -1, +1, +1, -1, -1, +1,<br>-1, +1, +1, -1, -1, +1, +1, -1, +1, -1, -1, +1, +1, -1, -1, +1 |

# 2 Regularized noise spectroscopy

## 2.1 Impact of electronic noise

The regularized noise spectroscopy approach was tested by generating DDNS input $\boldsymbol{\chi}$ = -ln($\boldsymbol{W}$) from a series of simulated noise spectra **S**. Upon adding white noise $\boldsymbol{N}$ of amplitude $N$ to model an experimental decoherence vector $\boldsymbol{W}$' = $\boldsymbol{W}$+$\boldsymbol{N}$, noise spectrum reconstruction failed to obtain the true underlying $\boldsymbol{S}$ even for a very small noise amplitude $N = 0.001$ which corresponds to a standard deviation of $2.9 \cdot 10^{-4}$ (Fig. S1). This observation arises from the logarithmized decoherence vector that enters the regularization expression (equation (3), main text) as input with $\boldsymbol{\chi} = -\ln(\boldsymbol{W}+\boldsymbol{N}) = \boldsymbol{\chi} - \ln(1+\boldsymbol{N}/\boldsymbol{W})$. Therefore, the deviation of $\boldsymbol{\chi}$' from $\boldsymbol{\chi}$ depends on both $N$ and $\boldsymbol{W}$. More specifically, $\boldsymbol{\chi}$' converges to –ln($N$) according to

$$\boldsymbol{\chi}' = -\ln(\boldsymbol{N}+\boldsymbol{W}) = -\ln(N) - \ln\left(1+\frac{\boldsymbol{W}}{\boldsymbol{N}}\right) \tag{S1}$$

as the second summand approaches zero (Fig. S1(b), (d)). Modeling $\boldsymbol{W}$ with the SE expression demonstrates that the time point $T$ at which the noise amplitude dominates the tail of $\boldsymbol{W}$ depends on $T_m$, $\xi$ and $N$ (Fig. S1(b), (d)). For this reason, experimental DD data cannot serve as input to

regularization-based DDNS, and a functional decoherence description is needed instead. We apply adaptive wavelet denoising to obtain ***W*** by fitting ***W'*** to the SE or SSE (equation (4), main text) model, which serve as a low-pass filter. The applied fitting procedure is described in the SI of [2].

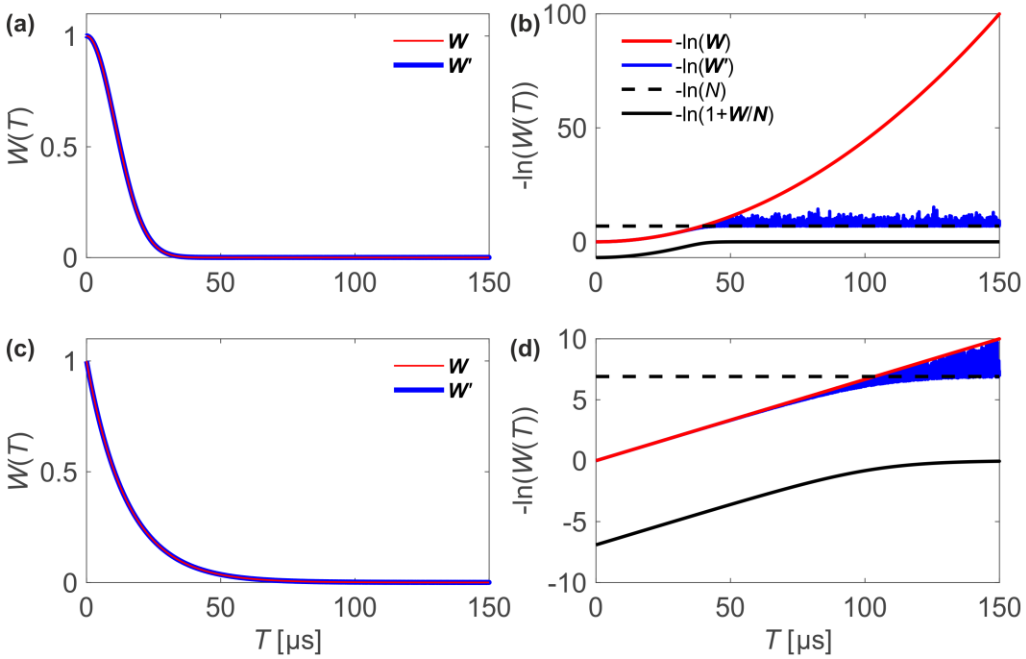


**Fig. S1 Influence of electronic noise in the detected EPR signal onto DDNS.** Two simulated decoherence vectors ***W*** (red) modelled using the SE expression with $T_m$ = 15 µs, (a) $\xi = 2$ and (c) $\xi = 1$. The corresponding ***W'*** (blue) is obtained after adding white noise with amplitude $N$ = 0.001. (b) Logarithmic representation for $\xi = 2$, where the $-\ln(N)$ level (black, dashed) is shown. The second summand of equation (S1) (black, solid) converges towards zero. (d) Same representation for $\xi = 1$.

## 2.2 Determining the accessible frequency range

As described in the main text the experimental decoherence vector ***W'*** determines the accessible frequency range of the noise spectrum extending from $\omega_{min}$ to $\omega_{max}$. The first and last detectable element $W'_1 = W'(T_1)$ and $W'n_T = W'(Tn_T)$ define $\omega_{max}$ and $\omega_{min}$, respectively through the kernel matrix elements $F_{1,j}$ and $Fn_{T,j}$. A Gaussian fit (with mean $\mu$ and standard deviation $\sigma$) to the main band pass feature defines $\omega_{max} = \mu+3\sigma$ (Fig. S2) and $\omega_{min} = \mu-3\sigma$ (Fig. S3). This procedure underestimates $\omega_{min}$ for the Hahn experiment (Fig. S3(b), black circle), and it is advantageous to determine $\omega_{min}(n = 1)$ from extrapolation (Fig. S3(b), black star) of a polynomial fit to $\omega_{min}(n$ = 2-5).

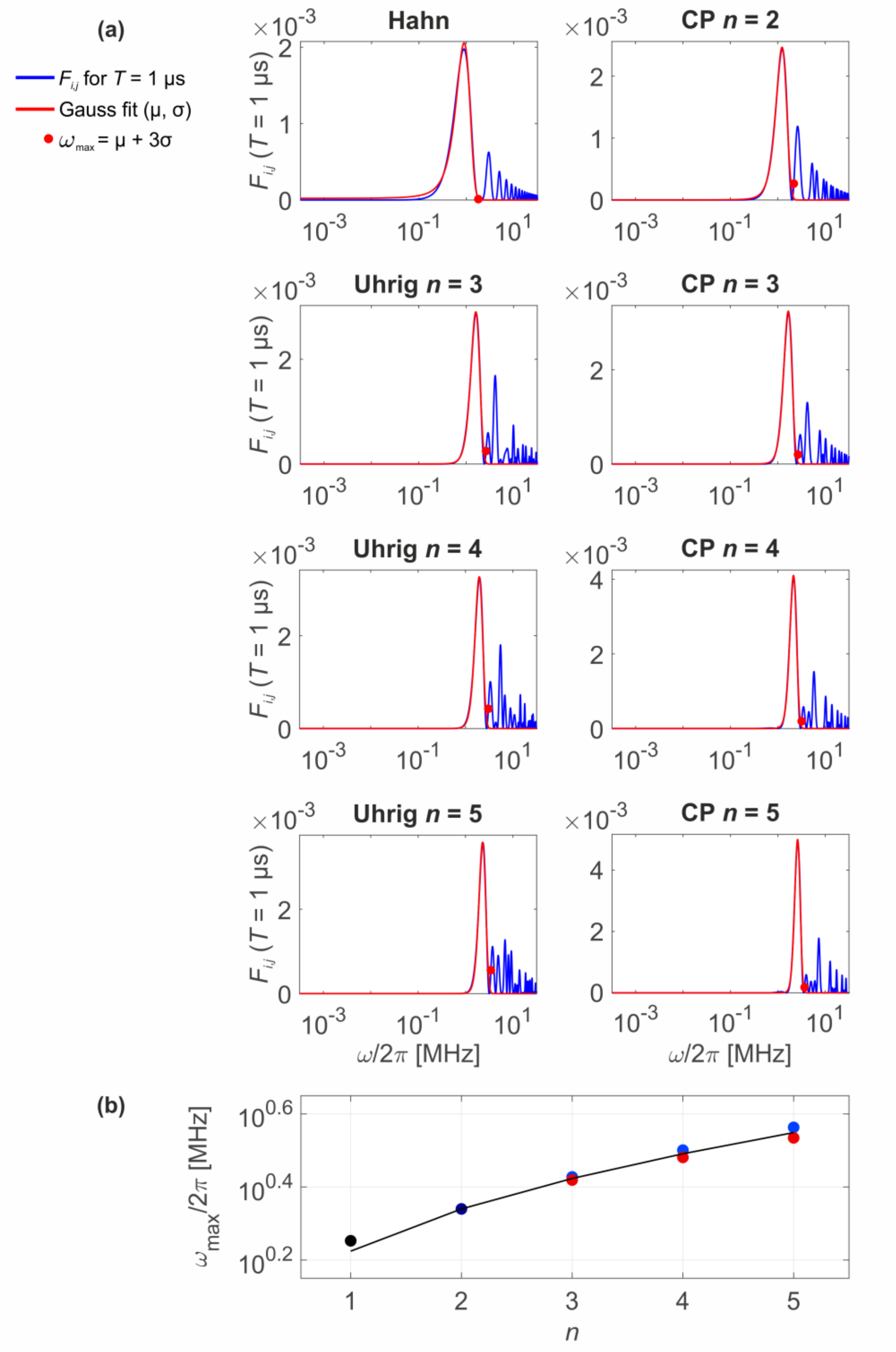


**Fig. S2 Maximal accessible frequency range ($\omega_{max}$) for DDNS determined from filter functions.** (a) Kernel matrix elements (blue) for the shortest considered sequence length $T_1$ = 1 μs to illustrate how $\omega_{max}$ is determined. A Gaussian fit (red) with mean $\mu$ and standard deviation σ to the main band pass feature of the filter kernel element determines $\omega_{max} = \mu + 3\sigma$, (b) resulting in a $\omega_{max}$ dependence on $n$ for $T_1$ = 1 μs.

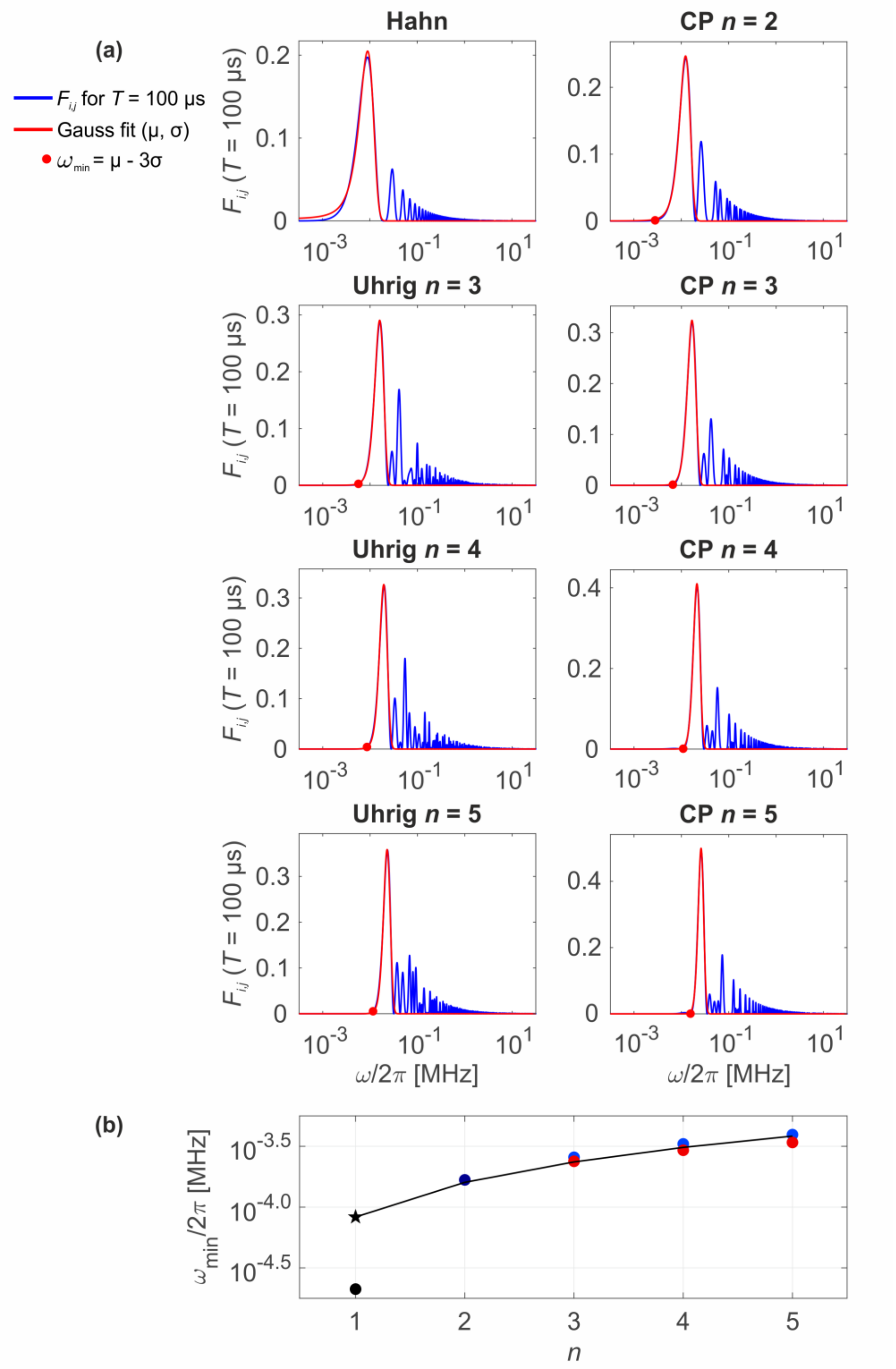


**Fig. S3 Minimal accessible frequency range ($\omega_{min}$) for DDNS determined from filter functions.** (a) Kernel matrix elements (blue) for an exemplary value of $Tn_T$ = 100 μs. A Gaussian fit (red) with mean $\mu$ and standard deviation $\sigma$ to the main band pass feature of the filter kernel element determines $\omega_{min} = \mu - 3\sigma$, (b) resulting in a $\omega_{min}$ dependence on $n$ for $Tn_T$ = 100 μs. This approach underestimates $\omega_{min}$ for the Hahn experiment (black circle), instead polynomial extrapolation to $\omega_{min}(n = 1)$ is used (black star).

All noise spectra models presented in the main text were obtained from regularized DDNS using a logarithmically spaced frequency vector. For low temperature DD data sets (at 40 K) the determined $\boldsymbol{S}_{med}$ and $\boldsymbol{S}_{IQR}$ overlap with solutions obtained from regularized DDNS with a linear distribution of $n_\omega$ elements across the frequency range ($\omega_{min}$-$\omega_{max}$). At intermediate (80 K) and high (298 K) temperatures the two approaches lead to noise spectra that differ in their width, as illustrated for 20 µM H-mNOHex in OTP at 80 K (Fig. S4). This deviation is likely to arise from different weighting of the noise spectra contribution at higher frequencies for a log(ω) and linear ω vector. In the latter case, a second peak is visible in the noise spectra for $n$ = 1-3 that appears due to spectral leakage away from the main pass band of the filter function (e.g. see filter kernel component beyond Gaussian fit in Fig. S2-3).

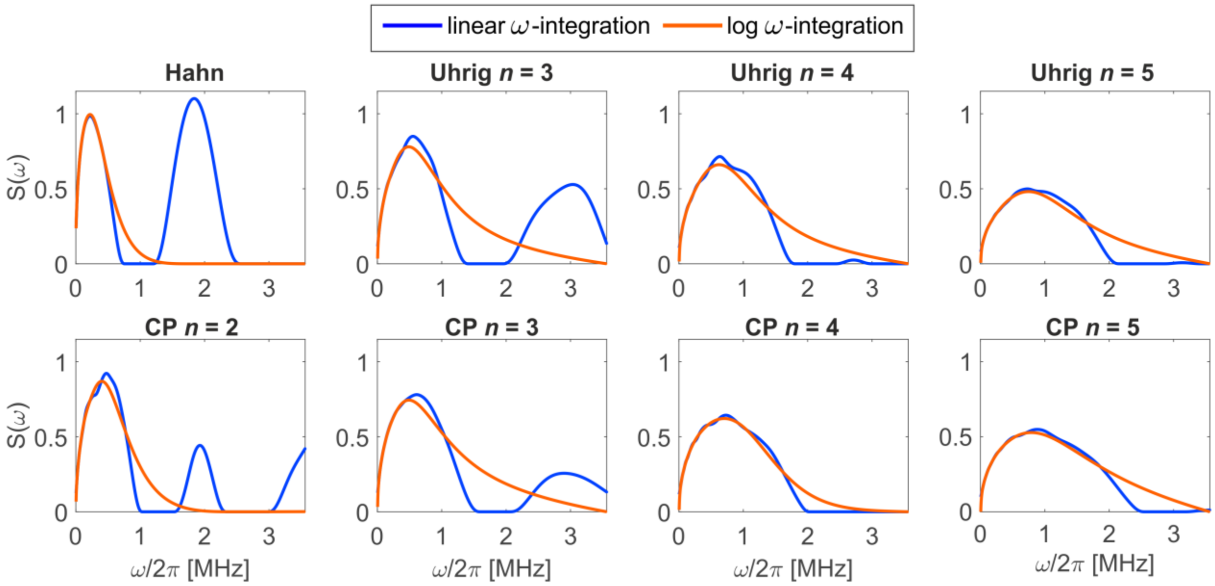


**Fig. S4 Case of deviation between DDNS results obtained with linear and logarithmic ω axis.** Experiment-specific noise spectra for 20 µM H-mNOHex in OTP at 80 K using a linear (blue) and logarithmic (orange) $n_\omega$ distribution of 500 points per 10 MHz of $\omega_{max}$-$\omega_{min}$.

## 2.3 DDNS-based decoherence description

The main text includes an illustrative example for the agreement of the DDNS-based decoherence description with the experimental DD traces of 20 µM D-mNOHex in dOTP at 40 K (Fig. 3). Figures S5 and S6 show the DDNS-based decoherence description for all other *o*-terphenyl and Fig. S7 for the water-glycerol noise spectra.

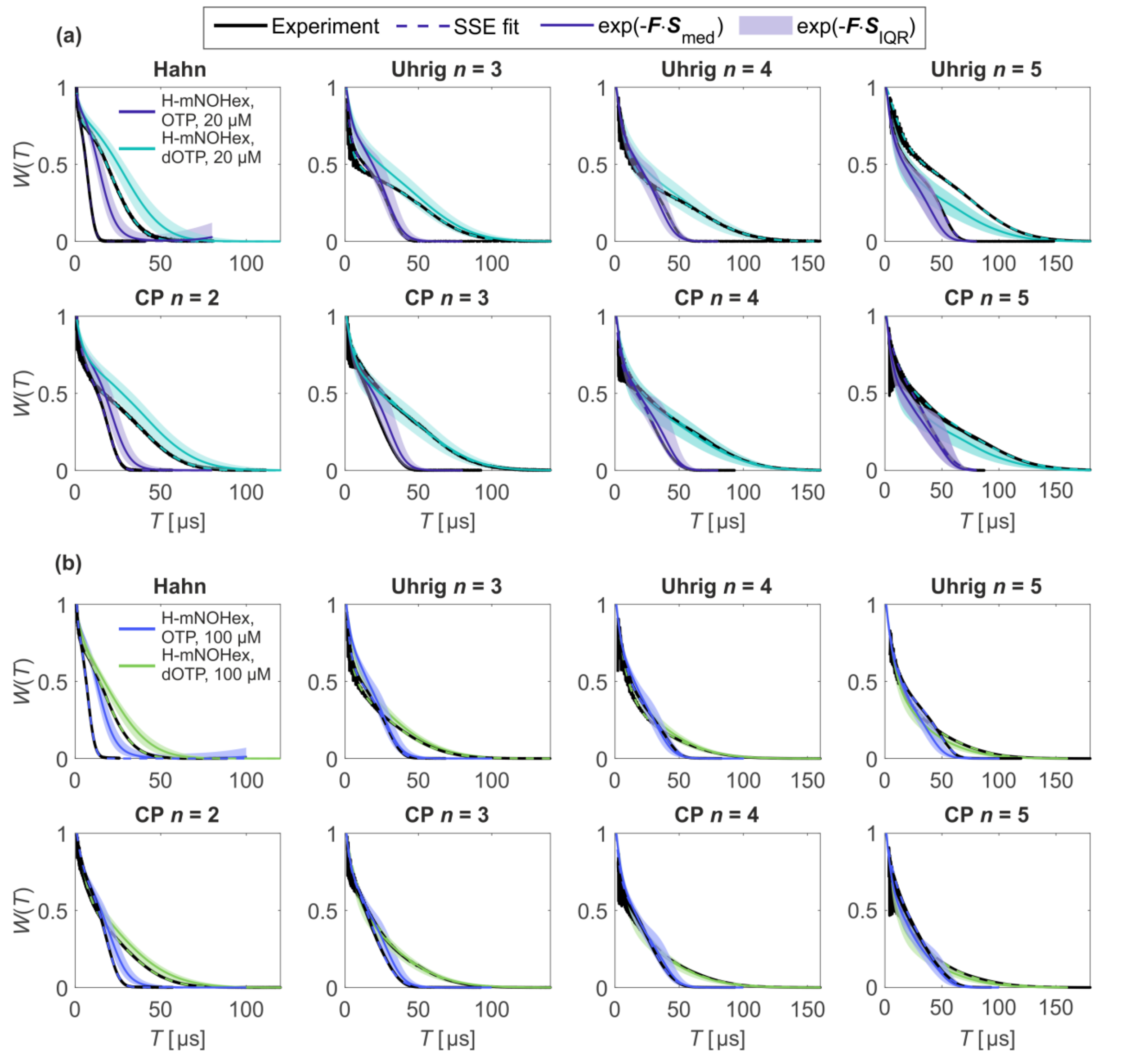


**Fig. S5 DDNS-based decoherence description for nitroxides in *o*-terphenyl glass at 40 K.** Measured decoherence (black) for DD experiments with $n$ = 1-5 for (a) 20 uM H-mNOHex in OTP and dOTP and (b) 100 μM H-mNOHex in OTP and dOTP. The initial signal oscillation stems from nuclear modulation. DDNS using the SSE parameterization (colored, dashed line) generate a set of experiment-specific noise spectra. The median noise spectrum $S_{med}$ and the associated interquartile range $S_{IQR}$ (see Fig. 4(a)-(b)) provide a global DDNS-based decoherence description using exp(-F·$S_{med}$) (colored, solid line) and exp(-F·$S_{IQR}$) (colored, shaded area). Note the legend on top.

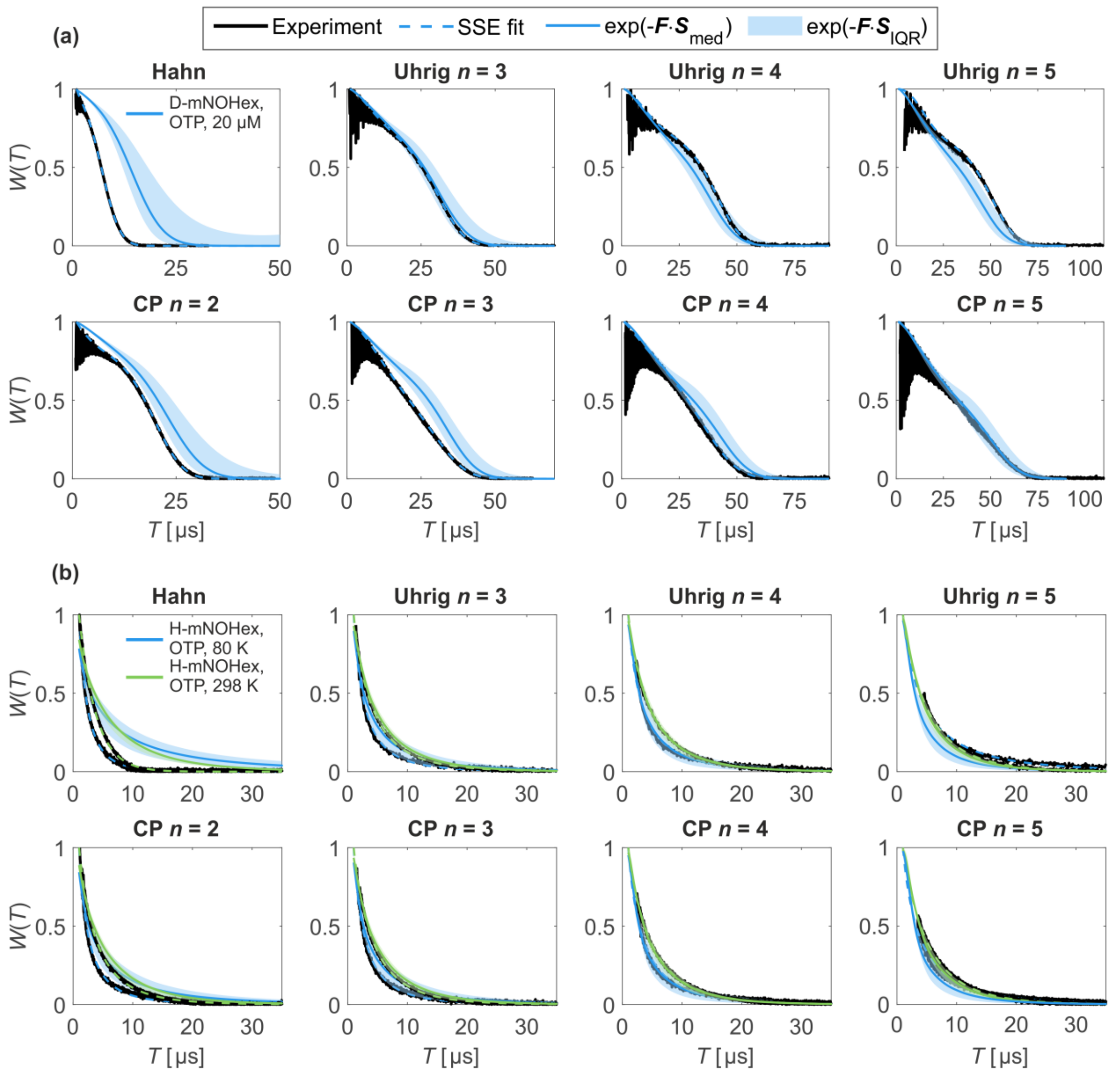


**Fig. S6 DDNS-based decoherence description for nitroxides in *o*-terphenyl glass at 40, 80 and 298 K.** Measured decoherence (black) for DD experiments with *n* = 1-5 for (a) 20 uM D-mNOHex in dOTP at 40 K and (b) 20 µM H-mNOHex in OTP at 80 and 298 K. The initial signal oscillation stems from nuclear modulation. DDNS using the SSE parameterization (colored, dashed line) generate a set of experiment-specific noise spectra. The median noise spectrum $S_{med}$ and the associated interquartile range $S_{IQR}$ (see Fig. 4(a)-(b) at 40 K and Fig. 6(b) for 80 and 298 K) provide a global DDNS-based decoherence description using $\exp(-F\cdot S_{med})$ (colored, solid line) and $\exp(-F\cdot S_{IQR})$ (colored, shaded area). Note the legend on top.

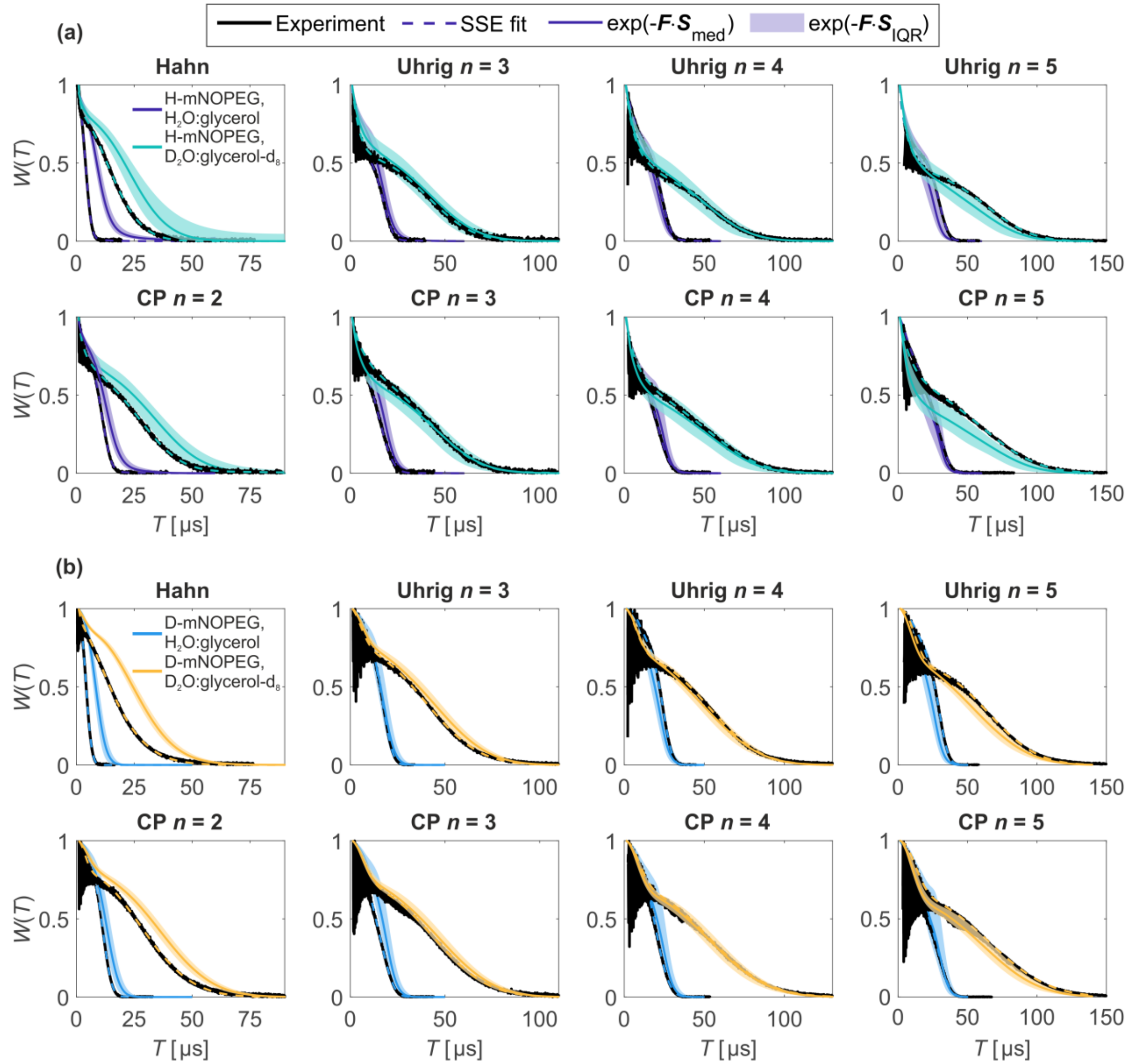


**Fig. S7 DDNS-based decoherence description for nitroxides in *o*-terphenyl glass at 40 K.** Measured decoherence (black) for DD experiments with $n$ = 1-5 for (a) 10 uM H-mNOPEG in $H_2O$:glycerol and $D_2O$:glycerol-$d_8$ and (b) 10 uM D-mNOPEG in $H_2O$:glycerol and $D_2O$:glycerol-$d_8$. The initial signal oscillation stems from nuclear modulation. DDNS using the SSE parameterization (colored, dashed line) generate a set of experiment-specific noise spectra. The median noise spectrum $S_{med}$ and the associated interquartile range $S_{IQR}$ (see Fig. 5(a)-(b)) provide a global DDNS-based decoherence description using $\exp(-F{\cdot}S_{med})$ (colored, solid line) and $\exp(-F{\cdot}S_{IQR})$ (colored, shaded area). Note the legend on top.

# 3 Static solid-state $^1$H NMR spectra

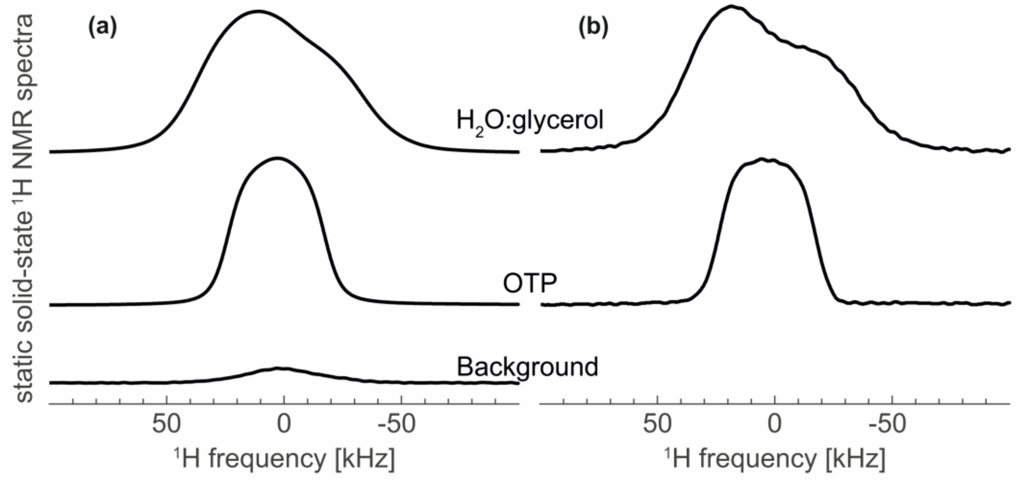


**Fig. S8 Background correction of static solid-state $^1$H NMR spectra.** Static solid-state $^1$H NMR solid echo spectra of $H_2O$:glycerol and OTP in the absence of radical (a) before and (b) after subtraction of the background spectrum, acquired at 14.1 T and 100 K. For background subtraction, the spectral intensities were normalized to the same number of scans.

## 4 Tunnel ESEEM simulations

### 4.1 2pESEEM/Hahn sequence

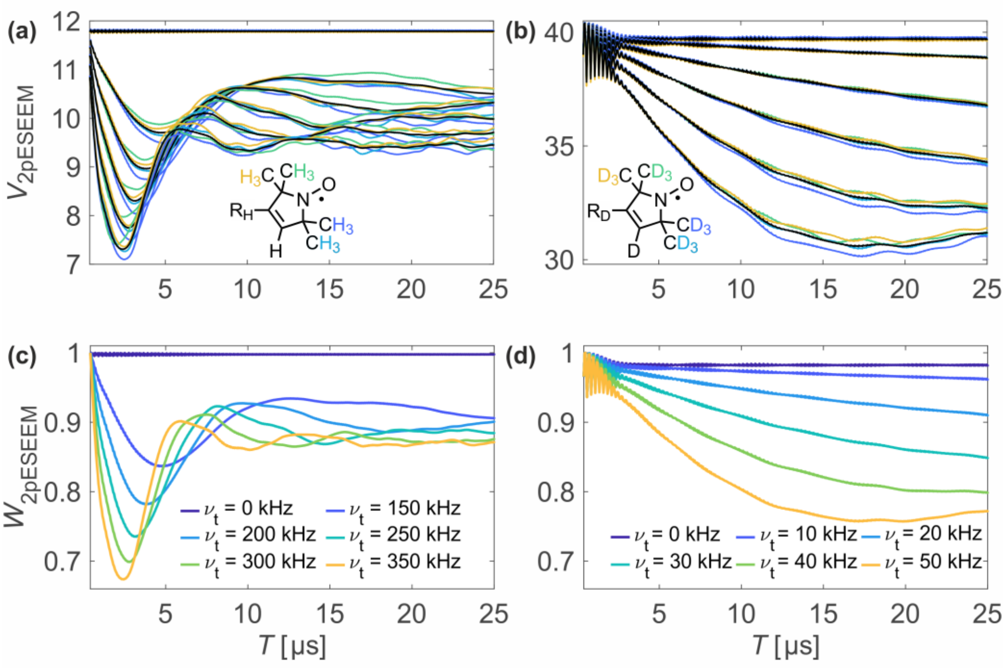


**Fig. S9 Two-pulse ESEEM simulations.** Simulated electron spin coherence under the two-pulse ESEEM/Hahn experiment by density operator formalism for a nitroxide featuring (a, c) protonated and deuterated (b, d) methyl groups. Absolute (a-b) and normalized (c-d) two-pulse ESEEM traces obtained after powder averaging over 31 orientations as a function of tunnel frequency $\nu_t$. (a-b) show individual contributions from the four methyl groups (color code see inset) along with the averaged trace (black), displayed after normalization in (c-d). For deuterated methyl groups the nuclear quadrupole interaction was taken into account with $\nu_{NQ}$ = 200 kHz.

### 4.2 CP $n$ = 2-4 sequence

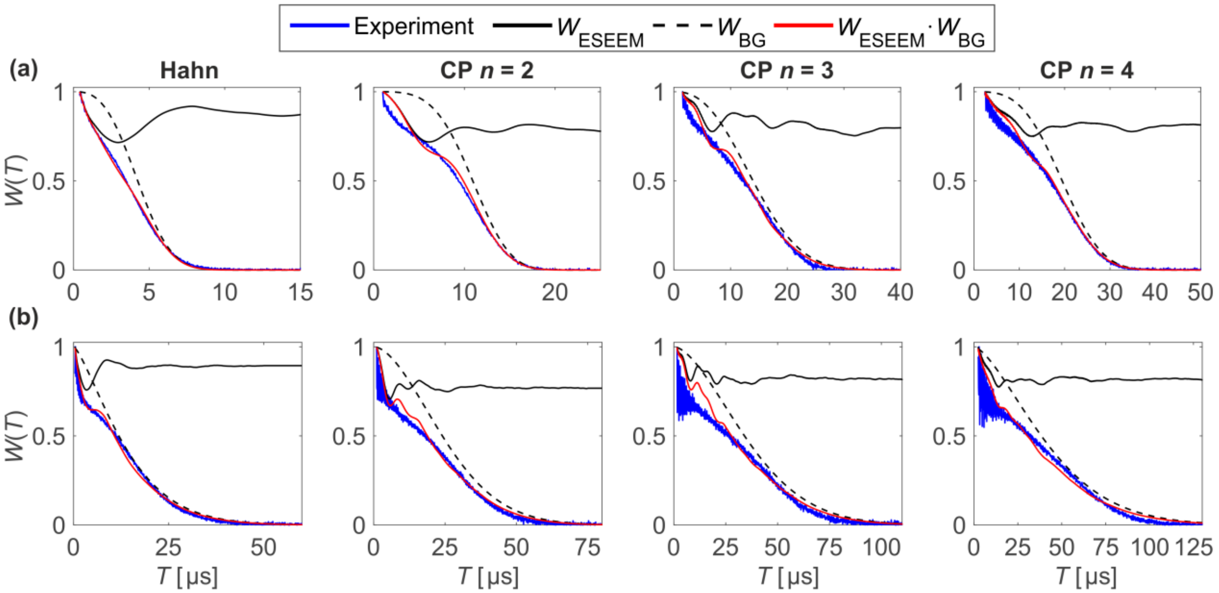


**Figure S10. Tunnel frequency estimation from DD measurements with CP $n$ = 1-4.** DD measurements (for $n$ = 1 to 4 from left to right) of 10 µM H-mNOPEG in (a) $H_2O$:glycerol and (b) $D_2O$:glycerol-$d_8$ at 40 K (blue). The tunnel frequency is obtained from fits (red) with the product of the background function $W_{BG}$, modelled by an SE expression with $c$ = 1, (black, dashed) and the simulated $\nu_t$-dependent quantum rotor ESEEM under the Hahn, CP $n$ = 2, 3 or 4 experiment (black, solid). Determined $\nu_t$ and $W_{BG}$ parameters $T_m$ and $\xi$ (a) Hahn: $\nu_t$ = 275 kHz, $T_m$ = 4.8 µs and $\xi$ = 2.80, CP $n$ = 2: $\nu_t$ = 250 kHz, $T_m$ = 12.0 µs and $\xi$ = 3.85, CP $n$ = 3: $\nu_t$ = 325 kHz, $T_m$ = 16.2 µs and $\xi$ = 2.45 and CP $n$ = 4: $\nu_t$ = 225 kHz, $T_m$ = 21.8 µs and $\xi$ = 3.25. (b) Hahn: $\nu_t$ = 225 kHz, $T_m$ = 15.6 µs and $\xi$ = 1.35, CP $n$ = 2: $\nu_t$ = 275 kHz, $T_m$ = 29.6 µs and $\xi$ = 1.80, CP $n$ = 3: $\nu_t$ = 275 kHz, $T_m$ = 42.4 µs and $\xi$ = 1.70 and CP $n$ = 4: $\nu_t$ = 200 kHz, $T_m$ = 49.0 µs and $\xi$ = 1.50.

# 5 References


1 J. Soetbeer, M. Hülsmann, A. Godt, Y. Polyhach and G. Jeschke, Dynamical decoupling of nitroxides in o-terphenyl: a study of temperature, deuteration and concentration effects, *Phys. Chem. Chem. Phys.*, 2018, **20**, 1615–1628.

2 J. Soetbeer, M. Millen, K. Zouboulis, M. Hülsmann, A. Godt, Y. Polyhach and G. Jeschke, Dynamical decoupling in water–glycerol glasses: a comparison of nitroxides, trityl radicals and gadolinium complexes, *Phys. Chem. Chem. Phys.*, 2021, **23**, 5352–5369.
